# 2-(2-Phenylethyl)chromone Derivatives of Agarwood Originating from *Gyrinops salicifolia*

**DOI:** 10.3390/molecules21101313

**Published:** 2016-10-03

**Authors:** Hang Shao, Wen-Li Mei, Wen-Hua Dong, Cui-Juan Gai, Wei Li, Guo-Peng Zhu, Hao-Fu Dai

**Affiliations:** 1 Horticultural and Garden College, Hainan University, Haikou 570228, Hainan, China; shaohang126@163.com; 2 Key Laboratory of Biology and Genetic Resources of Tropical Crops, Ministry of Agriculture, Institute of Tropical Bioscience and Biotechnology, Chinese Academy of Tropical Agricultural Sciences, Haikou 571101, Hainan, China; meiwenli@itbb.org.cn (W.-L.M.); dongwenhua@itbb.org.cn (W.-H.D.); gaicuijuan@163.com (C.-J.G.); liwei@itbb.org.cn (W.L.)

**Keywords:** 2-(2-phenylethyl)chromone derivative, 2-(2-phenylethenyl)chromone derivative, agarwood, *Gyrinops salicifolia*, cytotoxicity

## Abstract

Three new 2-(2-phenylethyl)chromone derivatives (**1**–**3**) and a new 2-(2-phenylethenyl)chromone derivative (**4**), together with two known 2-(2-phenylethyl)chromone derivatives (**5**–**6**), were isolated from agarwood originating from *Gyrinops salicifolia* Ridl. The structures of compounds **1**–**4** were elucidated by comprehensive spectroscopic techniques (UV, IR, 1D and 2D-NMR) and MS analysis, as well as by comparison with the literature. Compounds **1**, **2**, and **5** showed moderate cytotoxicity against human tumor K562, BEL-7402, and SGC-7901 cell lines with IC_50_ values of 5.76 to 20.1 µM.

## 1. Introduction

Agarwood (Chen-Xiang in Chinese) is the resinous heartwood of the plants from the *Aquilaria* or *Gyrinops* genus that belong to the family of Thymelaeaceae [1]. It is well known as a traditional medicinal and natural perfume material, and has become more and more prevalent in international trade [2,3]. As traditional medicine, agarwood can alleviate stomachaches and ease symptoms of cough, rheumatism and high fever. Furthermore, its special fragrance is able to calm people down and relieve fatigue [4,5]. Agarwood formation occurs slowly and infrequently in nature and the supply of wild agarwood cannot meet the market demand, so the studies on fragrant constituents and related biosynthetic genes [5,6,7,8,9], as well as the search for wild agarwood-producing species [10], become critical.

**Figure 1:**
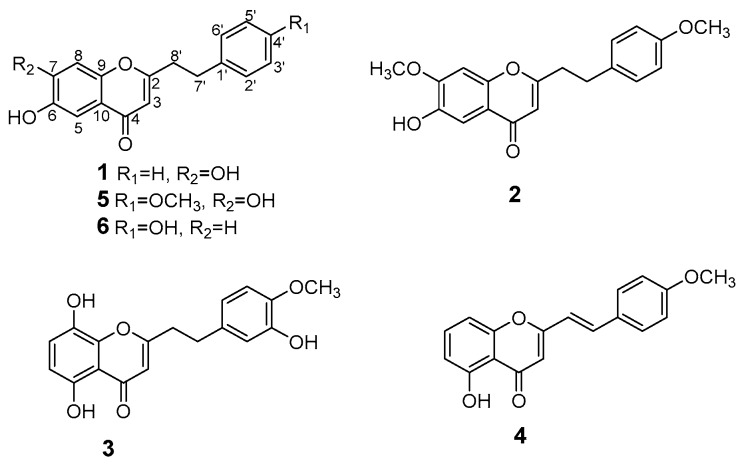
Chemical structure of compounds **1**–**6**.

*Gyrinops salicifolia*, currently listed in CITES Appendix II [11], is one of the agarwood-producing endemic species in Papua New Guinea. Previous studies on agarwood mainly focused on the resins obtained from *Aquilaria* species [5,6], although *Gyrinops* species also produce agarwood. As far as we know, the only report on chemical constituents of *Gyrinops* species is on the leaves and stems of *Gyrinops* *walla* [12]. It is worth noting that the chemical constituents of resins from *Gyrinops walla* identified by GC-MS were chemically similar to those from *Aquilaria* sp. [13]*.* So far, it has been reported that 2-(2-phenylethyl)chromone derivatives and sesquiterpenes were the main chemical constituents of agarwood [5,6,14,15]. Our study on chemical constituents of the agarwood from *Gyrinops salicifolia* led to isolation of three new 2-(2-phenylethyl)chromone derivatives (**1**–**3**) and a new 2-(2-phenylethenyl)chromone derivative (**4**), together with two known chromone derivatives (**5**,**6**) (Figure 1). In this paper, the isolation and structure elucidation of compounds **1**–**6** as well as cytotoxic activity against human tumor K562, BEL-7402, SGC-7901 cell lines is described.

## 2. Results and Discussion

**Figure 2:**
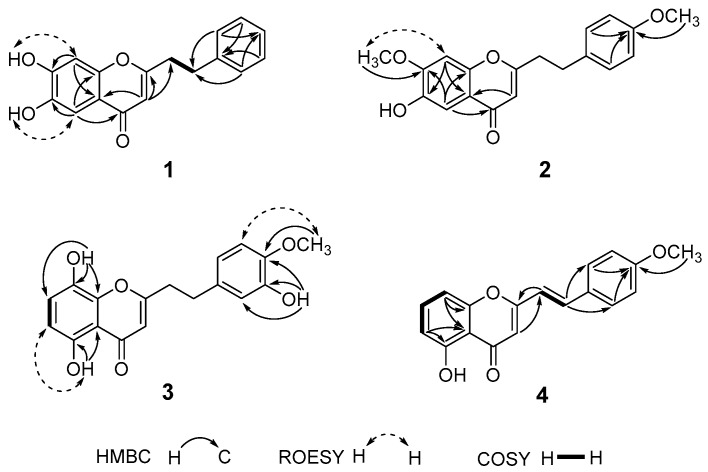
Key HMBC, ROESY, and ^1^H-^1^H COSY correlations of compounds **1**–**4**.

Compound **1** was obtained as a jacinth powder. Its molecular formula was deduced to be C_17_H_14_O_4_ on the basis of HR-ESI-MS (*m/z* 281.0820 [M − H]^−^). The IR spectrum revealed the presence of hydroxyl (3434 cm^−1^) and α,β-unsaturated carbonyl (1632 cm^−1^) functionalities. The ^1^H-NMR (Table 1, Supplementary) spectrum displayed two hydroxyl proton resonances at δ_H_ 10.35 (1H, s, 7-OH) and 9.70 (1H, s, 6-OH), two *para*-position aromatic protons at δ_H_ 7.22 (1H, s, H-5) and 6.84 (1H, s, H-8), five characteristic protons on the mono-substituted aromatic ring at δ_H_ 7.26 (4H, m, H-2′, 3′, 5′, 6′) and δ_H_ 7.19 (1H, m, H-4′), one olefinic proton at δ_H_ 5.99 (1H, s, H-3), as well as four methylene protons at δ_H_ 2.97 (2H, m, H_2_-7′) and δ_H_ 2.90 (2H, m, H_2_-8′) which connected to each other according to the ^1^H-^1^H COSY correlation from H_2_-7′ to H_2_-8′ (Figure 2). The ^13^C-NMR (Table 1) and HSQC spectra showed that compound **1** contained a chromone nucleus by the signals at δ_C_ 107.6 (C-5), 144.3 (C-6), 152.0 (C-7), 102.8 (C-8), 115.8 (C-10) and 151.0 (C-9), as well as an α,β-unsaturated carbonyl moiety by signals at δ_C_ 167.2 (C-2), 108.6 (C-3) and 176.0 (C-4). Additional signals included a mono-substituted aromatic ring (C-1′ (δ_C_ 140.2), C-2′/6′ (δ_C_ 128.4), C-3′/5′ (δ_C_ 128.3), C-4′ (δ_C_ 126.2)) and two methylene carbons at δ_C_ 32.1 (C-7′) and 34.7 (C-8′). These data were quite similar to those of compound **5** [16], with the main difference observed being the replacement of signals for the 4-methoxyphenyl unit in **5** by signals for the phenyl unit in **1**. It can be further confirmed by the observed HMBC correlation from H-5 (δ_H_ 7.22) to C-4 (δ_C_ 176.0) which was much stronger than that from H-8 (δ_H_ 6.84) to C-4, indicating that δ_H_ 7.22 and δ_H_ 6.84 were assigned to H-5 and H-8, respectively (Figure 2). Thus, the structure of **1** was assigned as 6,7-dihydroxy-2-(2-phenylethyl)chromone.

Compound **2** was obtained as a colorless oil. Its molecular formula was deduced to be C_19_H_18_O_5_ on the basis of HR-ESI-MS data (*m/z* 327.1224 [M + H]^+^). The ^1^H-NMR spectrum showed the presence of two methoxy groups at δ_H_ 3.89 (7-OCH_3_) and δ_H_ 3.70 (4′-OCH_3_), one hydroxyl group at δ_H_ 9.72 (6-OH), one set of protons on the AA′BB′ coupling system at δ_H_ 7.15 (2H, d, *J* = 8.5 Hz, H-2′, 6′) and δ_H_ 6.83 (2H, d, *J* = 8.5 Hz, H-3′, 5′), as well as two singlet aromatic protons at δ_H_ 7.23 (1H, s, H-5) and 7.12 (1H, s, H-8). The ^1^H- and ^13^C-NMR data of **2** closely resembled that of known compound 6-hydroxy-7-methoxy-2-(2-phenylethyl)chromone [17], except for an additional methoxy group in **2**. These also can be confirmed by the HMBC correlation of OCH_3_ (δ_H_ 3.89) to C-7 (δ_C_ 153.6), and its ROESY correlation with H-8 (δ_H_ 7.12). The position of an additional methoxy group at C-4′ was determined by the HMBC correlations from 4′-OCH_3_ (δ_H_ 3.70), H-2′/H-6′ (δ_H_ 7.15) and H-3′/H-5′ (δ_H_ 6.83) to the same quaternary carbon C-4′ (δ_C_ 157.8) (Figure 2). Thus, the structure of **2** was proposed to be 6-hydroxy-7-methoxy-2-[2-(4-methoxyphenyl)ethyl]chromone.

Compound **3** was obtained as a yellow powder. Its molecular formula was deduced to be C_18_H_16_O_6_ on the basis of HR-ESI-MS (*m/z* 327.0873 [M − H]^−^). The ^1^H-NMR spectrum showed the presence of a methoxy group at δ_H_ 3.71 (4′-OCH_3_), three hydroxyl groups at δ_H_ 9.67 (8-OH), 11.85 (5-OH) and 8.85 (3′-OH), one set of signals with two *ortho*-coupled doublets at δ_H_ 6.61 (1H, d, *J* = 8.2 Hz, H-6) and δ_H_ 7.17 (1H, d, *J* = 8.2 Hz, H-7), and another set of ABX coupling aromatic system signals at δ_H_ 6.61 (1H, dd, *J* = 8.2, 2.2 Hz, H-6′), δ_H_ 6.67 (1H, d, *J* = 2.2 Hz, H-2′) and δ_H_ 6.80 (1H, d, *J* = 8.2 Hz, H-5′). The comparison of NMR data between **3** and 5,8-dihydroxy-2-[2-(4-methoxyphenyl)ethyl]chromone [18] suggested that their structures were closely related, except that an additional hydroxy group was located at C-3′ in **3**. HMBC correlations from the hydroxy group at δ_H_ 8.85 to C-4′ (δ_C_ 146.4), C-3′ (δ_C_ 146.2) and C-2′ (δ_C_ 115.7) (Figure 2) further corroborated this deduction. Thus, **3** was deduced to be 5,8-dihydroxy-2-[2-(3-hydroxy-4-methoxyphenyl)ethyl]chromone.

Compound **4** was obtained as a yellow powder, and its molecular formula was deduced to be C_18_H_16_O_4_ on the basis of HR-ESI-MS (*m/z* 293.1787 [M − H]^−^). The IR spectrum indicated the presence of a hydroxyl (3435 cm^−1^) and an α,β-unsaturated carbonyl (1631 cm^−1^) group. Its ^1^H-NMR spectroscopic data showed two *trans*-olefinic protons at δ_H_ 7.59 (1H, d, *J* = 16.0 Hz, H-7′) and δ_H_ 6.63 (1H, d, *J* = 16.0 Hz, H-8′), a set of AA′BB′ coupling aromatic systems at δ_H_ 7.54 (2H, d, *J* = 8.1 Hz, H-2′, 6′) and δ_H_ 6.95 (2H, d, *J* = 8.1 Hz, H-3′, 5′), three mutual coupled aromatic protons at δ_H_ 6.79 (1H, d, *J* = 8.3 Hz, H-6), δ_H_ 7.51 (1H, t, *J* = 8.3 Hz, H-7) and δ_H_ 6.96 (1H, d, *J* = 8.3 Hz, H-8) corresponding to a 1,2,3-substituted phenyl moiety in the chromone nucleus, and a methoxy group at δ_H_ 3.86 (3H, s, OCH_3_-4′). The ^1^H- and ^13^C-NMR spectroscopic data of **4** showed a high degree of similarity with those of 5-hydroxy-2-(2-phenyletheyl)chromone [19]. The main difference observed was that signals for the *trans* double-bond (δ_H_ 7.59 and δ_H_ 6.63) in **4** replaced the corresponding signals for two vicinal methylenes in 5-hydroxy-2-(2-phenyletheyl)chromone, and the presence of one methoxy signal in **4**. Finally, compound **4** was established to be 5-hydroxy-2-[2-(4-methoxyphenyl)ethenyl]chromone by a comprehensive analysis of its 2D-NMR data (Figure 2).

The structures of compounds **5**–**6**, which have been isolated from agarwood, were identified as 6,7-dihydroxy-2-[2-(4-methoxyphenyl)ethyl]chromone (**5**) [16] and 6-hydroxy-2-[2-(4-hydroxyphenyl)ethyl]chromone (**6**) [17], respectively, by comparison of the-NMR and MS data with those reported by literature.

All isolated compounds were evaluated for their cytotoxic activity toward K562, BEL-7402, and SGC-7901 human cancer cell lines using the MTT assay, with paclitaxel as a positive control. For K562 and BEL-7402 cell lines, compound **5** showed relatively pronounced cytotoxicity with IC_50_ values of 8.36 and 5.76 µM. However, demethoxylation on C-4′ in compound **1** caused a significant decrease in the activity against the above cell lines with IC_50_ values of 18.1 and 20.1 µM, respectively. Both compounds **1** and **5** were inactive against the SGC-7901 cell line. Notably, compound **2**, with a methoxyl substituted at C-7 instead of a hydroxyl as in compound **5**, was active to all three cell lines, including moderate cytotoxicity against SGC-7901 and K562 cells with IC_50_ values of 17.8 and 13.9 µM, respectively, and weak cytotoxicity against BEL-7402 cells with an IC_50_ value of 31.9 µM. Compared with compound **5**, compound **6** harbored a hydroxyl linked at C-4′ and no substituent at C-7, and only showed weak cytotoxicity against K562 cells with an IC_50_ value of 47.0 µM. Compounds **3** and **4** were inactive against the aforementioned cell lines.

According to the results, we found that compounds **1**, **2**, **5** and **6** showed more effective cytotoxic activity to K-562 cells than to BEL-7402 and SGC-7901 cell lines. In addition, a comprehensive comparison of the cytotoxic activity of compounds **1**–**6** suggested that hydroxyls substituted at C-6 and C-7, respectively, as in **1** and **5**, led to more potent activity, while **5** with methoxy at C-4′ showed increased activity. Furthermore, the hydroxy group at C-6 was crucial for the cytotoxic activities among this type of compound.

## 3. Experimental Section

### 3.1. General Information

UV spectra were recorded on a Shimadzu UV-2550 spectrometer (Beckman, Brea, CA, USA). IR absorptions were obtained on a Nicolet 380 FT-IR instrument (Thermo, Pittsburgh, PA, USA) using KBr pellets. The-NMR spectra were recorded on Bruker Avance 500-NMR spectrometers (Bruker, Bremen, Germany), using TMS as an internal standard. HR-ESI-MS were measured with an API QSTAR Pulsar mass spectrometer (Bruker, Bremen, Germany) or Waters Autospec Premier (Waters, Milford, MA, USA). Column chromatography was performed with silica gel (60–80, 200–300 mesh, Qingdao Haiyang Chemical Co., Ltd, Qingdao, China), ODS gel (20–45 µm, Fuji Silysia Chemi-cal Co., Ltd, Research Triangle Park, NC, USA), and Sephadex LH-20 (Merck, Darmstadt, Germany). TLC was carried out on silica gel GF254 (Qingdao Haiyang Chemical Co., Ltd, Qingdao, China), and spots were detected by spraying with 5% H_2_SO_4_ in EtOH followed by heating.

### 3.2. Plant Material

The plant material was collected in Papua New Guinea, then traded in Macao, China′s special administrative regions, in December 2014, and identified as agarwood originating from *Gyrinops salicifolia* by Prof. Dr. Hao-Fu Dai and Dr. Jun Wang (Institute of Tropical Bioscience and Biotechnology, Chinese Academy of Tropical Agricultural Sciences & Hainan engineering research center of agarwood). A voucher specimen (CX 20141222) has been deposited at the Institute of Tropical Bioscience and Biotechnology, Chinese Academy of Tropical Agricultural Sciences.

### 3.3. Extraction and Isolation

The material of agarwood (491.1 g, dry weight) was extracted with 95% EtOH (2 L × 3) for three times at heating reflux and filtered. Then the EtOH was removed under vacuum, and get crude extract 177.4 g. After that scatter it completely in H_2_O (2 L), subsequently extracted with EtOAc (2 L × 3) followed by *n*-BuOH (2 L × 3). The EtOAc extract (141.2 g) was subjected to vacuum liquid chromatography with silica gel using a step gradient of CHCl_3_/MeOH (*v/v*, 1:0, 50:1, 25:1, 15:1, 10:1, 5:1, 2:1, 1:1, 0:1, 6 L of each) to yield 10 fractions (Fr.1–Fr.10). Fr.3 (7.2 g) was applied to ODS column chromatography with MeOH/H_2_O (*v/v*, 3:7, 2:3, 1:1, 3:2, 7:3, 4:1, 9:1, 1:0, 2 L of each) divided to 19 fractions (Fr.3-1–Fr.3-19). Fr.3-3 (85.2 mg) was submitted to Sephadex LH-20 in MeOH to get Fr.3-3-1 (36.0 mg), then purified through silica gel column chromatography with CHCl_3_/MeOH (*v/v*, 100:1) to obtain compound **6** (4.3 mg). Fr.3-5 (143.4 mg) was applied to Sephadex LH-20 in MeOH to get Fr.3-5-1 (75.0 mg), then separated through silica gel column chromatography with CHCl_3_/MeOH (*v/v*, 100:1) to afford compound **1** (5.1 mg). Fr.3-6 (99.3 mg) was applied to Sephadex LH-20 in MeOH to get Fr.3-6-1 (66.0 mg), then compound **5** (18.0 mg) was obtained through silica gel column chromatography with CHCl_3_-MeOH (*v/v*, 100:1). Fr.3-10 (156.1 mg) was applied to Sephadex LH-20 (MeOH; CHCl_3_/MeOH, *v/v*, 1:1) to get Fr.3-10-1 (63.0 mg), then purified through silica gel column chromatography (petroleum ether/EtOAc, *v/v*, 40:1) to obtain compound **4** (2.0 mg). Fr.3-12 (62.6 mg) was applied to Sephadex LH-20 (MeOH), then purified through silica gel column chromatography (CHCl_3_/MeOH, *v/v*, 150:1) to obtain compound **3** (6.7 mg). Fr.3-14 (108.0 mg) was applied to Sephadex LH-20 (MeOH), then purified through silica gel column chromatography (CHCl_3_/MeOH, *v/v*, 500:1) to obtain compound **2** (14.0 mg).

*6,7-Dihydroxy-2-(2-phenylethyl)chromone* (**1**): jacinth powder; UV (MeOH) λ_max_ (log ε): 208 (4.69), 228 (4.58), 282 (4.22), 298 (4.08), 322 (4.25) nm; IR (KBr) *v*_max_ 3434, 1632, 1384, 697 cm^−1^; ^1^H- (500 MHz) and ^13^C- (125 MHz) NMR spectral data see Table 1; HR-ESI-MS: *m/z* 281.0820 [M − H]^−^ (calcd for C_17_H_14_O_4_, 282.0892).

*6-Hydroxy-7-methoxy-2-[2-(4-methoxyphenyl)ethyl]chromone* (**2**): colorless oil; UV (MeOH) λ_max_ (log ε): 230 (4.78), 280 (4.41), 298 (4.24), 322 (4.39) nm; IR (KBr) *v*_max_ 3404, 1639, 1385, 1017 cm^−1^; ^1^H- (500 MHz) and ^13^C- (125 MHz) NMR spectral data see Table 1; HR-ESI-MS: *m/z* 327.1224 [M + H]^+^ (calcd for C_19_H_18_O_5_, 326.1154).

*5,8-Dihydroxy-2-[2-(3-hydroxy-4-methoxyphenyl)ethyl]chromone* (**3**): yellow powder; UV (MeOH) λ_max_ (log ε): 204 (5.12), 224 (4.68), 256 (4.55), 296 (4.65), 320 (4.05) nm; IR (KBr) *v*_max_ 3467, 1634, 1015, 670 cm^−1^; ^1^H- (500 MHz) and ^13^C- (125 MHz) NMR spectral data see Table 1; HR-ESI-MS: *m/z* 327.0873 [M − H]^–^ (calcd for C_18_H_16_O_6_, 328.0947).

*5-Hydroxy-2-[2-(4-methoxyphenyl)ethenyl]chromone* (**4**): yellow powder; UV (MeOH) λ_max_ (log ε): 204 (5.04), 224 (4.56), 256 (4.15), 296 (3.92), 306 (3.73) nm; IR (KBr) *v*_max_ 3435, 1631, 1384, 772 cm^−1^; ^1^H- (500 MHz) and ^13^C- (125 MHz) NMR spectral data see Table 1; HR-ESI-MS: *m/z* 293.1787 [M − H]^–^ (calcd for C_18_H_16_O_4_, 294.1858).

### 3.4. Bioassay of Cytotoxic Activity

Human cancer cell lines, gastric carcinoma (SGC-7901), myeloid leukemia (K562), and hepatocellular carcinoma (BEL-7402), were obtained from the Cell Bank of Type Culture Collection of the Chinese Academy of Sciences, Shanghai Institute of Cell Biology. MTT assay [20,21] was used to determine the growth inhibition of the tested cell lines. Cells were cultured in RPMI 1640 medium supplemented with 10% fetal bovine serum, 100 IU/mL penicillin, and 100 mg/mL streptomycin at 37 °C and 5% CO_2_ with 90% humidity. The logarithmic phase cells (90 µL) were seeded onto 96-well plates at the concentration of 5 × 10^4^ cell/mL. The following specific experimental procedures were the same as those described previously [22]. The results of the cytotoxic activity experiment are shown in Table 2.

## 4. Conclusions

To the best of our knowledge, 2-(2-phenylethyl)chromone derivatives, the compounds with a chromone skeleton, such as **1**–**3**, **5** and **6** isolated from the agarwood of *Gyrinops salicifolia*, are very common in nature, and have been reported in agarwoods from *Aquilaria* species [5,16]. For 2-(2-phenylethenyl)chromone derivatives, only one compound, 6-hydroxy-2-[2-(4-hydroxy-3- methoxyphenyl)ethenyl]chromone [16], was found in agarwood from *Aquilaria sinensis*. Interestingly, compound **4**, discovered during this study, was the second 2-(2-phenylethenyl)chromone derivative from agarwood. Furthermore, eight known 2-(2-phenylethyl)chromone derivatives and a series of sesquiterpenes have been isolated from the agarwood of *Gyrinops salicifolia* in our previous study [23,24], and most of them have also been reported in agarwoods from *Aquilaria* sp. [5,6,16]. As we can see, the main chemical constituents of agarwood from *Gyrinops salicifolia* are similar to the agarwood resins from *Aquilaria* sp., so it can be considered that *Gyrinops salicifolia* is one of the commercial agarwood-producing species.

## Figures and Tables

**Table 1:** ^1^H- (500 MHz) and ^13^C- (125 MHz) NMR spectral data of compounds **1**–**4** (δ in ppm, *J* in Hz).

No.	1 ^a^		2 ^a^		3 ^a^		4 ^b^	
	δ_C_	δ_H_	δ_C_	δ_H_	δ_C_	δ_H_	δ_C_	δ_H_
2	167.2		167.8		170.8		163.6	
3	108.6	5.99 s	108.9	6.03 s	108.4	6.22 s	108.4	6.22 s
4	176.0		176.3		183.1		183.7	
5	107.6	7.22 s	107.3	7.23 s	151.2		161.5	
6	144.3		145.1		109.8	6.61 d (8.2), overlap	111.4	6.79 d (8.3)
7	152.0		153.6		121.8	7.17 d (8.2)	135.4	7.51 t (8.3)
8	102.8	6.84 s	100.4	7.12 s	137.6		106.9	6.96 d (8.3)
9	151.0		151.1		144.5		156.4	
10	115.8		116.6		110.4		111.1	
1′	140.2		132.1		132.5		127.7	
2′	128.4	7.26 m	129.4	7.15 d (8.5)	115.7	6.67 d (2.2)	129.6	7.54 d (8.1)
3′	128.3	7.26 m	113.9	6.83 d (8.5)	146.2		114.7	6.95 d (8.1)
4′	126.2	7.19 m	157.8		146.4		161.0	
5′	128.3	7.26 m	113.9	6.83 d (8.5)	112.3	6.80 d (8.2)	114.7	6.95 d (8.1)
6′	128.4	7.26 m	129.4	7.15 d (8.5)	118.8	6.61 d (2.2, 8.2), overlap	129.6	7.54 d (8.1)
7′	32.1	2.97 m	31.4	2.90 m	31.2	2.92 m	137.9	7.59 d (16.0)
8′	34.7	2.90 m	35.2	2.90 m	35.2	2.92 m	117.3	6.63 d (16.0)
5-OH						11.85 s		
6-OH		9.70 s		9.72 s				
7-OH		10.35 s						
8-OH						9.67 s		
3′-OH						8.85 s		
7-OCH_3_			56.3	3.89 s				
4′-OCH_3_			55.0	3.70 s	55.6	3.71 s	55.6	3.86 s

**^a^** Measured in DMSO-*d*_6_, **^b^** Measured in CDCl_3_.

**Table 2:** Cytotoxic activity of **1**–**6** against human tumor cell lines.

Compound	IC_50_ (μM)		
	SGC-7901	K-562	BEL-7402
**1**	>50	18.1	20.1
**2**	17.8	13.9	31.9
**3**	>50	>50	>50
**4**	>50	>50	>50
**5**	>50	8.36	5.76
**6**	>50	47.0	>50
Paclitaxel ^b^	1.80	7.20	2.40

^b^ positive control.

## Supplementary Materials

The following are available online at http://www.mdpi.com/1420-3049/21/10/1313/s1. The HR-ESI-MS,-NMR (1D and 2D) of compounds **1**–**4** are available as supporting information.
