# Supplementary material for: 2-(2-Phenylethyl)chromone Derivatives of Agarwood Originating from Gyrinops salicifolia
Source: Molecules. 2016 Oct 3;21(10):1313. doi: 10.3390/molecules21101313 (PMC6273548; doi:10.3390/molecules21101313)
Supplement: Supplementary file 1 [file molecules-21-01313-s001.pdf]

# Supplementary Materials: 2-(2-Phenylethyl)chromone Derivatives of Agarwood Originating from *Gyrinops salicifolia*

**Hang Shao, Wen-Li Mei, Wen-Hua Dong, Cui-Juan Gai, Wei Li, Guo-Peng Zhu and Hao-Fu Dai**

**Contents**

**Figure S1.** $^1$H-NMR spectrum (500 MHz) of compound **1** in DMSO.
**Figure S2.** $^{13}$C-NMR spectrum (125 MHz) of compound **1** in DMSO.
**Figure S3.** $^1$H-$^1$H COSY spectrum (500 MHz) of compound **1** in DMSO.
**Figure S4.** HSQC spectrum (500 MHz) of compound **1** in DMSO.
**Figure S5.** HMBC spectrum (500 MHz) of compound **1** in DMSO.
**Figure S6.** ROESY spectrum (400 MHz) of compound **1** in DMSO.
**Figure S7.** ESI(+)MS spectrum of compound **1**.
**Figure S8.** HRESI(+)MS spectrum of compound **1**.
**Figure S9.** $^1$H-NMR spectrum (500 MHz) of compound **2** in DMSO.
**Figure S10.** $^{13}$C-NMR spectrum (125 MHz) of compound **2** in DMSO.
**Figure S11.** $^1$H-$^1$H COSY spectrum (500 MHz) of compound **2** in DMSO.
**Figure S12.** HSQC spectrum (500 MHz) of compound **2** in DMSO.
**Figure S13.** HMBC spectrum (500 MHz) of compound **2** in DMSO.
**Figure S14.** ROESY spectrum (400 MHz) of compound **2** in DMSO.
**Figure S15.** ESI(+)MS spectrum of compound **2**.
**Figure S16.** HRESI(+)MS spectrum of compound **2**.
**Figure S17.** $^1$H-NMR spectrum (500 MHz) of compound **3** in DMSO.
**Figure S18.** $^{13}$C-NMR spectrum (125 MHz) of compound **3** in DMSO.
**Figure S19.** $^1$H-$^1$H COSY spectrum (500 MHz) of compound **3** in DMSO.
**Figure S20.** HSQC spectrum (500 MHz) of compound **3** in DMSO.
**Figure S21.** HMBC spectrum (500 MHz) of compound **3** in DMSO.
**Figure S22.** ROESY spectrum (400 MHz) of compound **3** in DMSO.
**Figure S23.** ESI(+)MS spectrum of compound **3**.
**Figure S24.** HRESI(+)MS spectrum of compound **3**.
**Figure S25.** $^1$H-NMR spectrum (500 MHz) of compound **4** in $CDCl_3$.
**Figure S26.** $^{13}$C-NMR spectrum (125 MHz) of compound **4** in $CDCl_3$.
**Figure S27.** $^1$H-$^1$H COSY spectrum (500 MHz) of compound **4** in $CDCl_3$.
**Figure S28.** HSQC spectrum (500 MHz) of compound **4** in $CDCl_3$.
**Figure S29.** HMBC spectrum (500 MHz) of compound **4** in $CDCl_3$.
**Figure S30.** ROESY spectrum (400 MHz) of compound **4** in $CDCl_3$.
**Figure S31.** ESI(+)MS spectrum of compound **4**.
**Figure S32.** HRESI(+)MS spectrum of compound **4**.

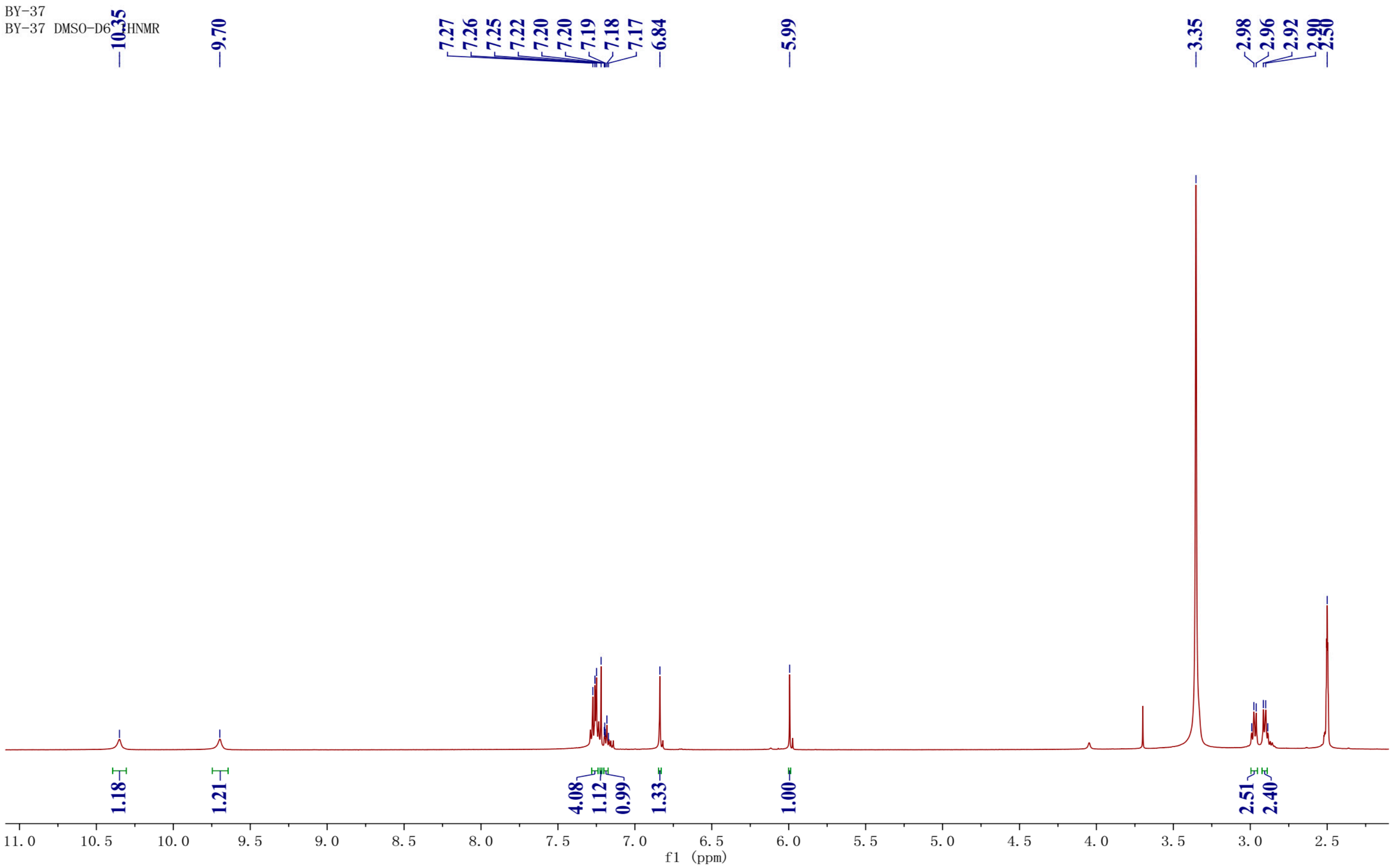


**Figure S1.** $^1$H-NMR spectrum (500 MHz) of compound **1** in DMSO.

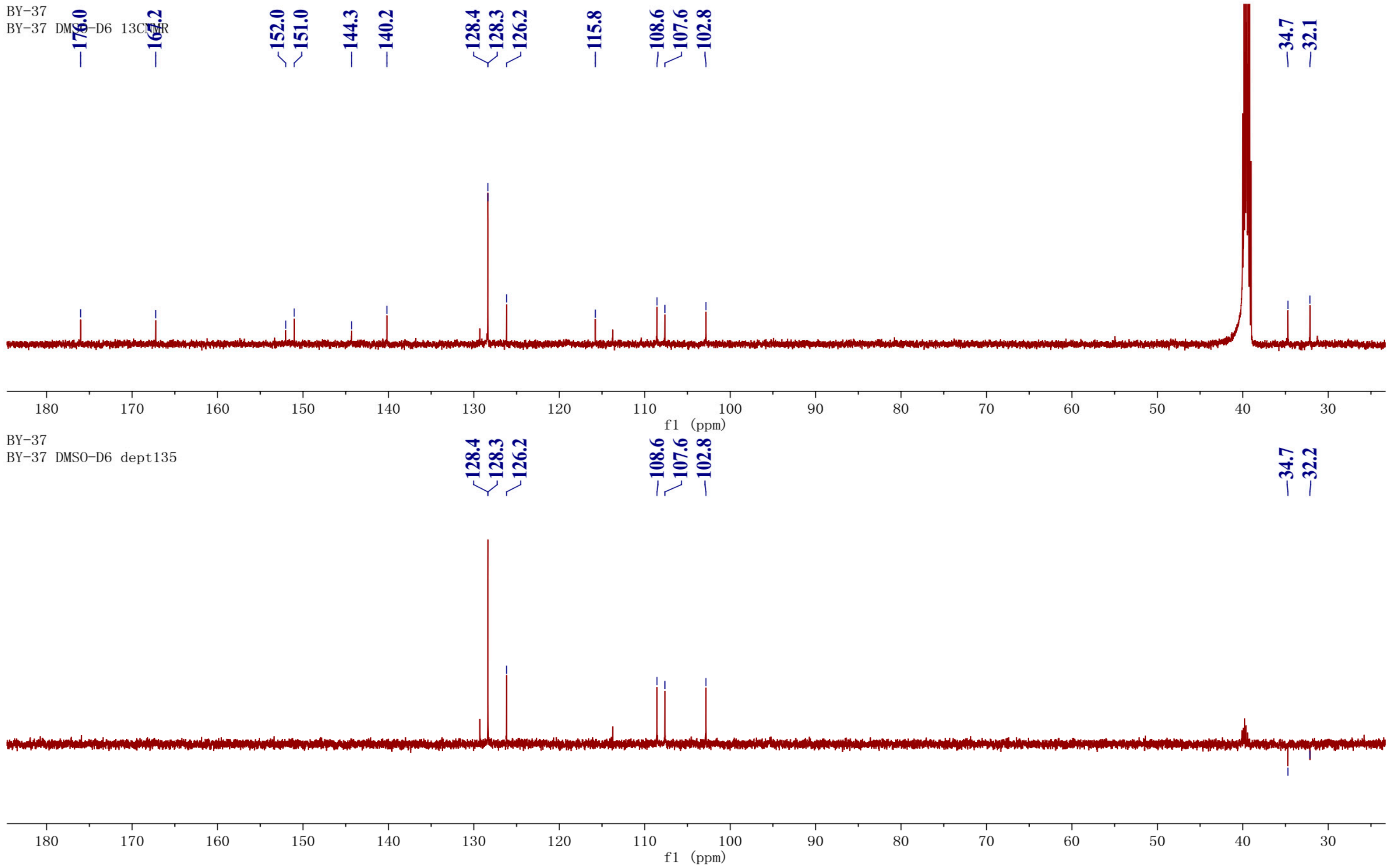


**Figure S2.** $^{13}$C-NMR spectrum (125 MHz) of compound **1** in DMSO.

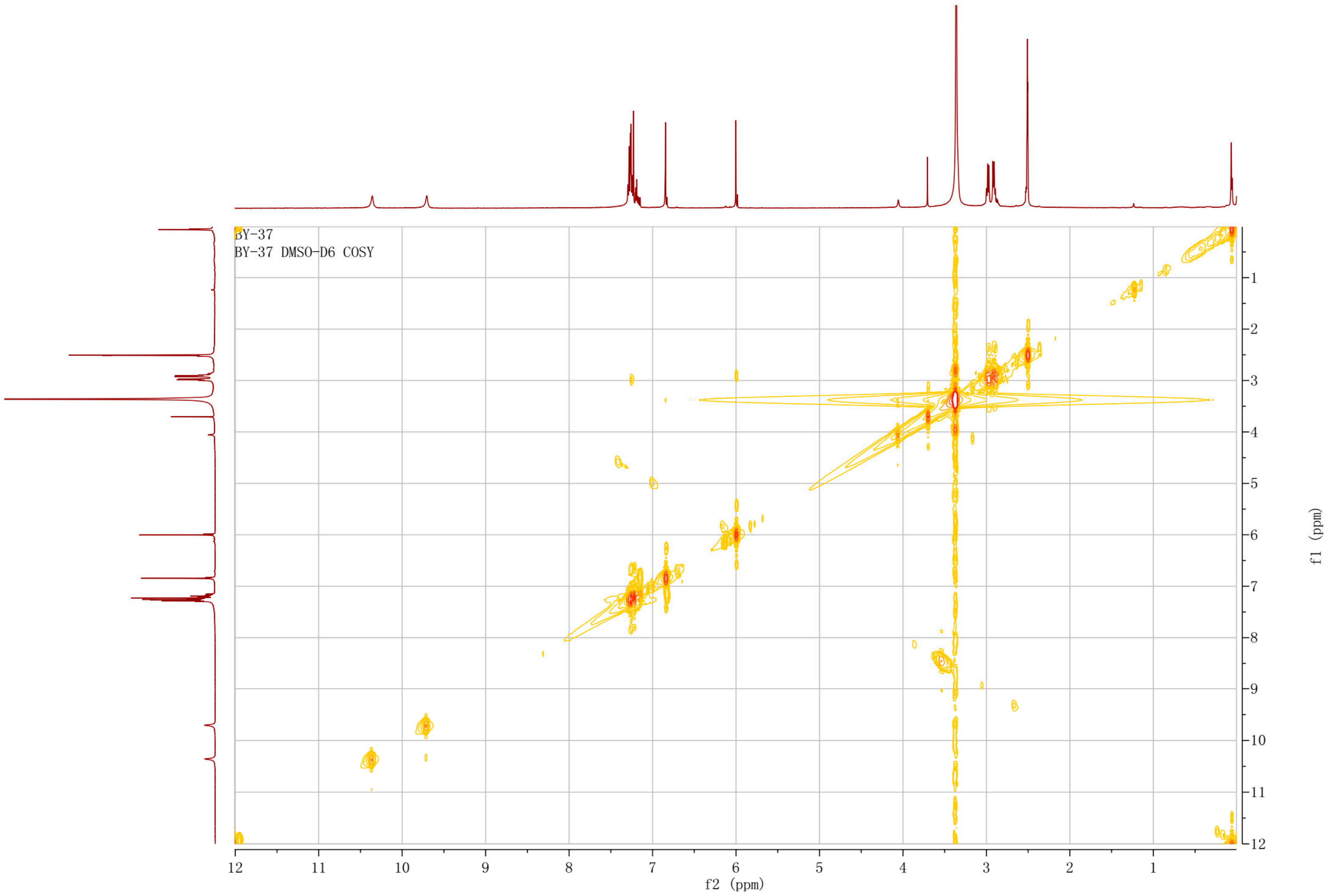


**Figure S3.** $^{1}$H-$^{1}$H COSY spectrum (500 MHz) of compound **1** in DMSO.

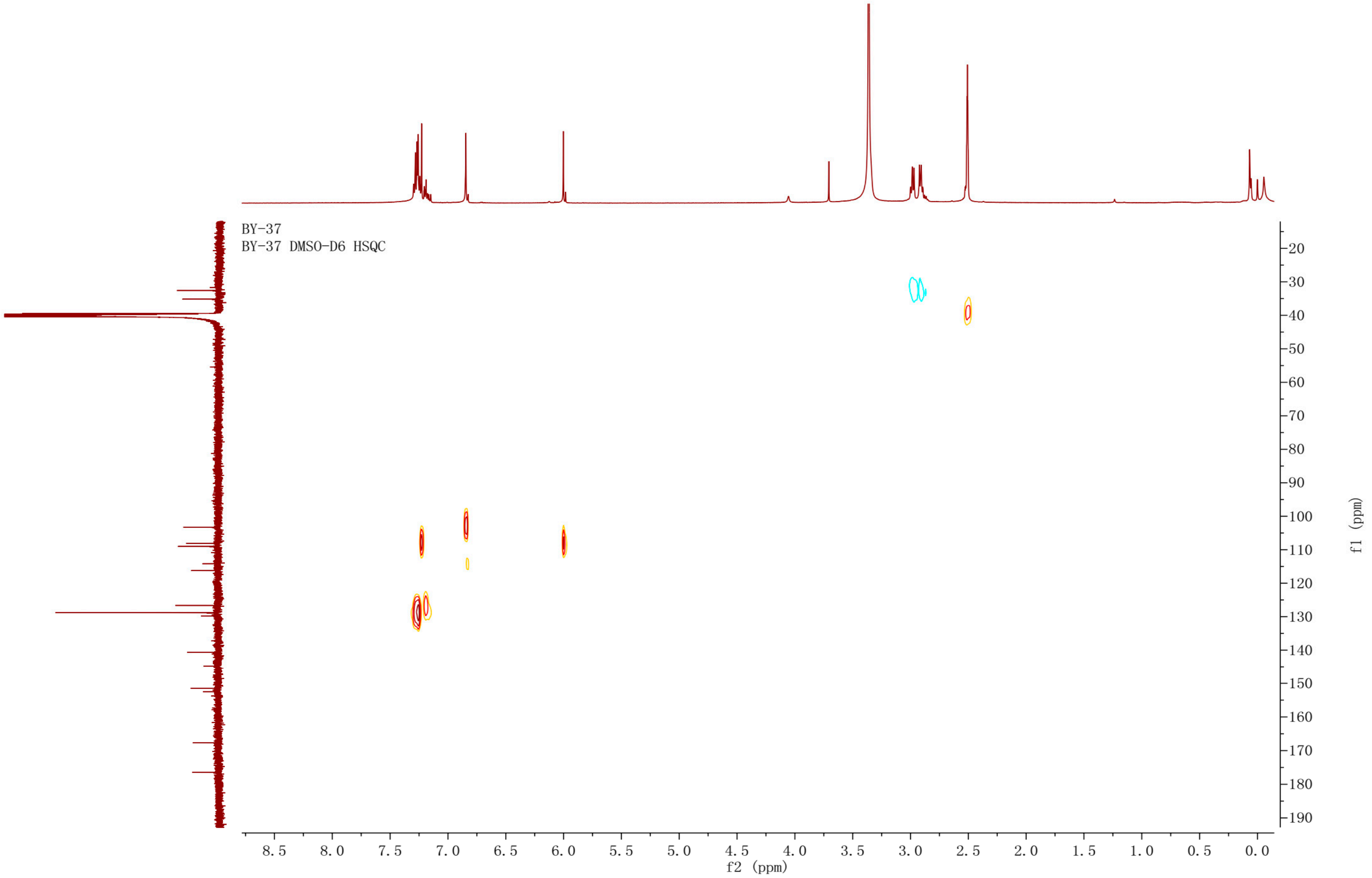


**Figure S4.** HSQC spectrum (500 MHz) of compound **1** in DMSO.

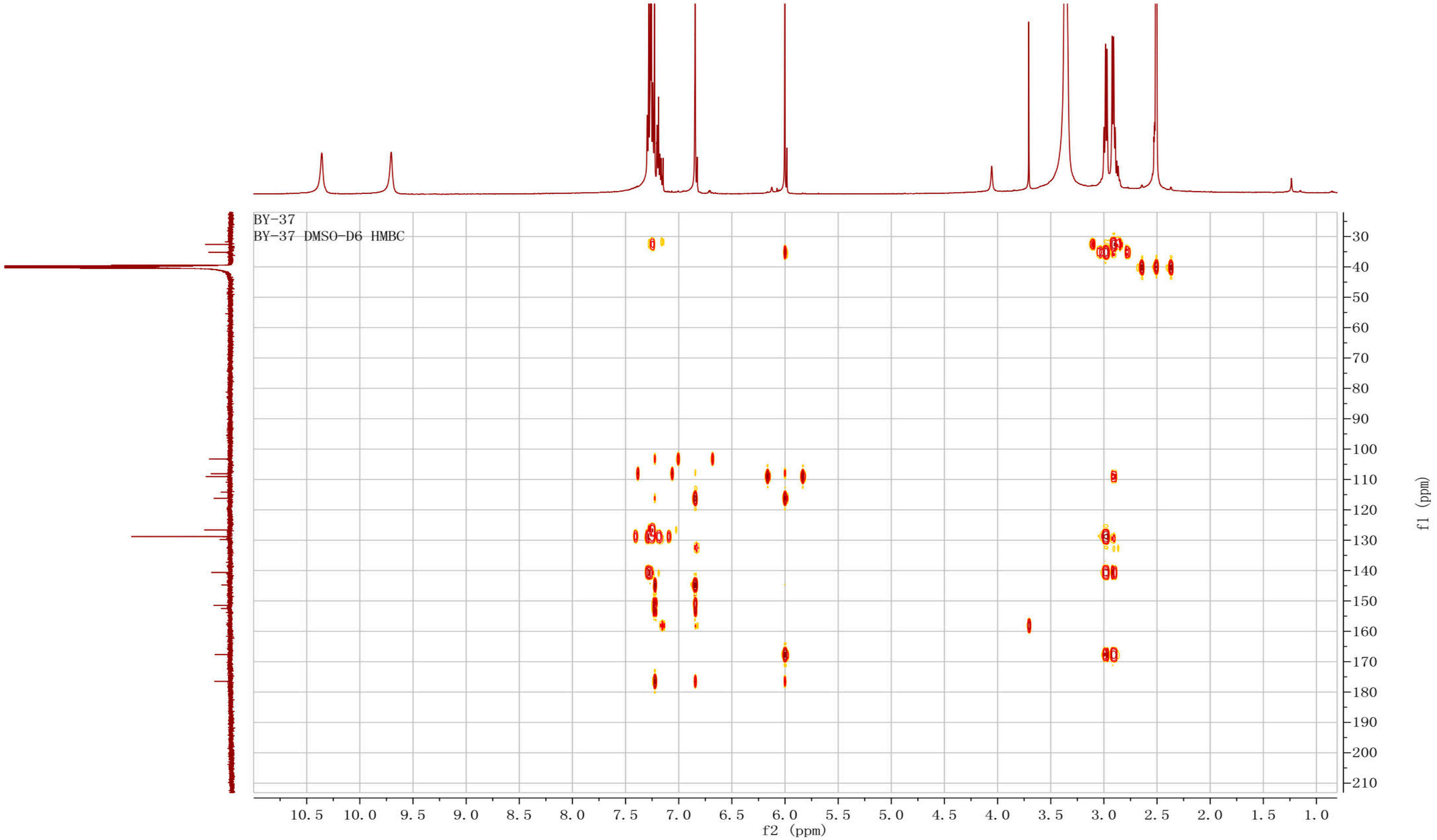


**Figure S5.** HMBC spectrum (500 MHz) of compound **1** in DMSO.

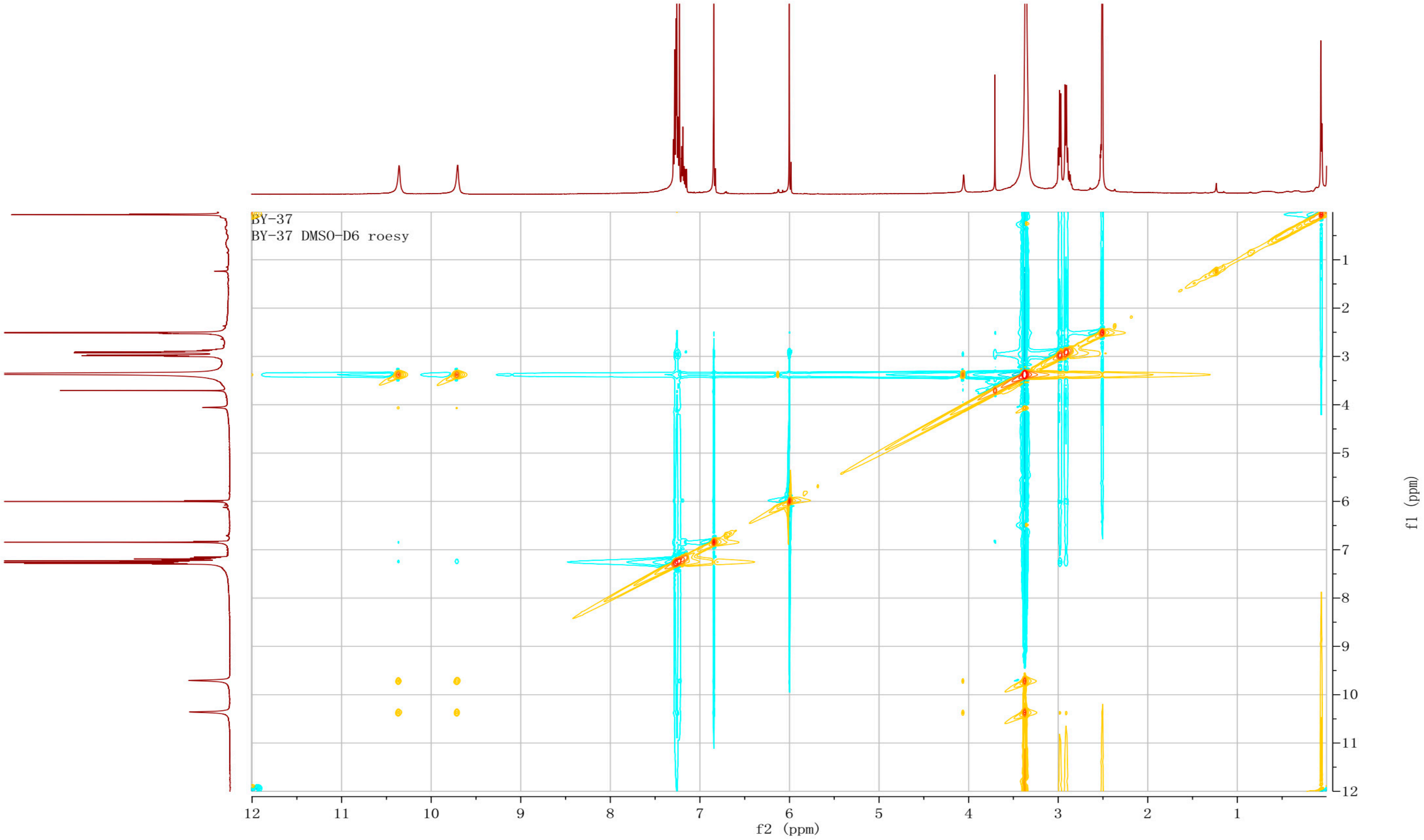


**Figure S6.** ROESY spectrum (400 MHz) of compound **1** in DMSO.

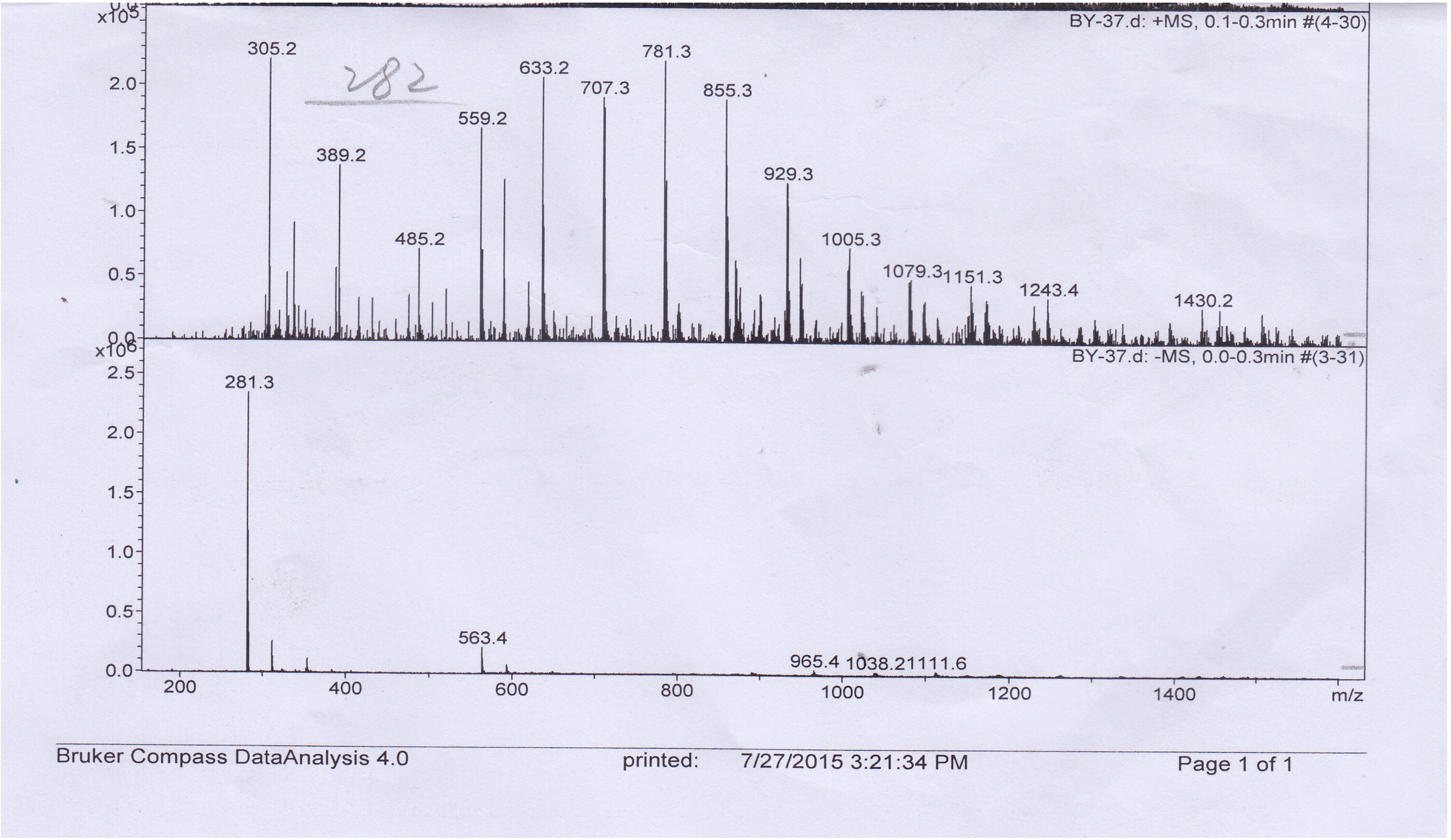


**Figure S7.** ESI(+)MS spectrum of compound **1**.

# Qualitative Analysis Report

| | | | |
|---|---|---|---|
| **Data Filename** | BY-37-.d | **Sample Name** | BY-37 |
| **Sample Type** | Sample | **Position** | P1-C1 |
| **Instrument Name** | Instrument 1 | **User Name** | |
| **Acq Method** | SIBU-ESI-i.m | **Acquired Time** | 10/22/2015 3:18:42 PM |
| **IRM Calibration Status** | Success | **DA Method** | ESI+.m |
| **Comment** | | | |

**Sample Group** **Info.**

**Acquisition SW** 6200 series TOF/6500 series
**Version** Q-TOF B.05.01 (B5125.2)

## User Spectra

| Fragmentor Voltage | Collision Energy | Ionization Mode |
|---|---|---|
| 135 | 0 | ESI |

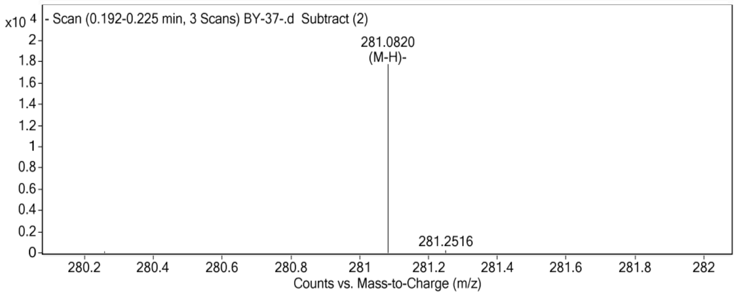


**Peak List**

| *m/z* | z | Abund | Formula | Ion |
|---|---|---|---|---|
| 281.082 | 1 | 17761.73 | C17 H14 O4 | (M-H)- |
| 282.0851 | 1 | 3844.94 | C17 H14 O4 | (M-H)- |
| 311.0921 | 1 | 4198.39 | | |
| 395.0744 | 1 | 31143.69 | | |
| 396.0783 | 1 | 6990.37 | | |
| 397.0799 | 1 | 1059.28 | | |
| 425.0846 | 1 | 10681.58 | | |
| 426.0884 | 1 | 2398.21 | | |

**Formula Calculator Element Limits**

| Element | Min | Max |
|---|---|---|
| C | 3 | 60 |
| H | 0 | 120 |
| O | 0 | 30 |
| N | 0 | 5 |

**Formula Calculator Results**

| Formula | CalculatedMass | CalculatedMz | Mz | Diff. (mDa) | Diff. (ppm) | DBE |
|---|---|---|---|---|---|---|
| C17 H14 O4 | 282.0892 | 281.0819 | 281.0820 | 0.0 | -0.1 | 11.0000 |

--- End Of Report ---



**Figure S8.** HRESI(+)MS spectrum of compound **1**.

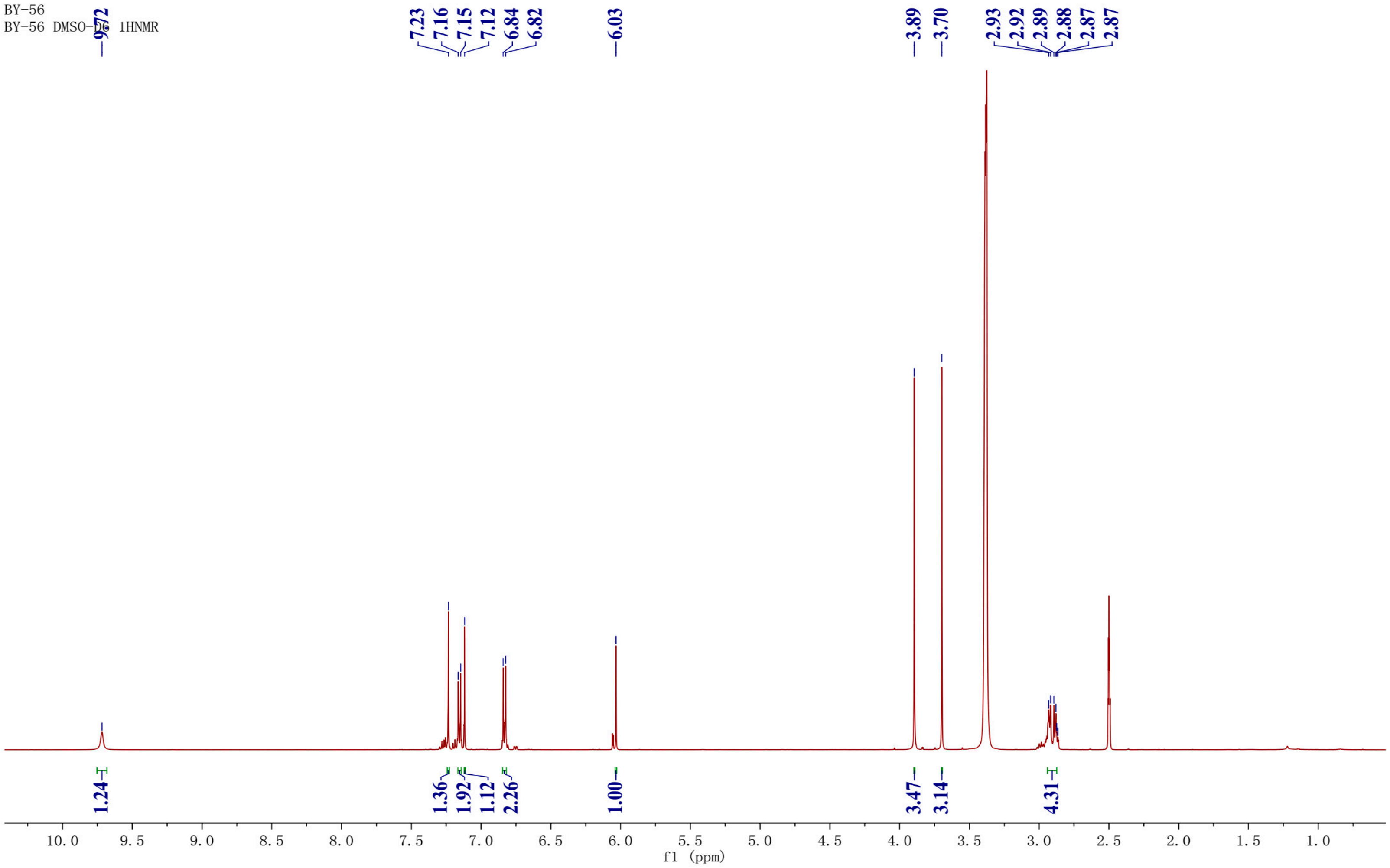


**Figure S9.** $^1$H-NMR spectrum (500 MHz) of compound **2** in DMSO.

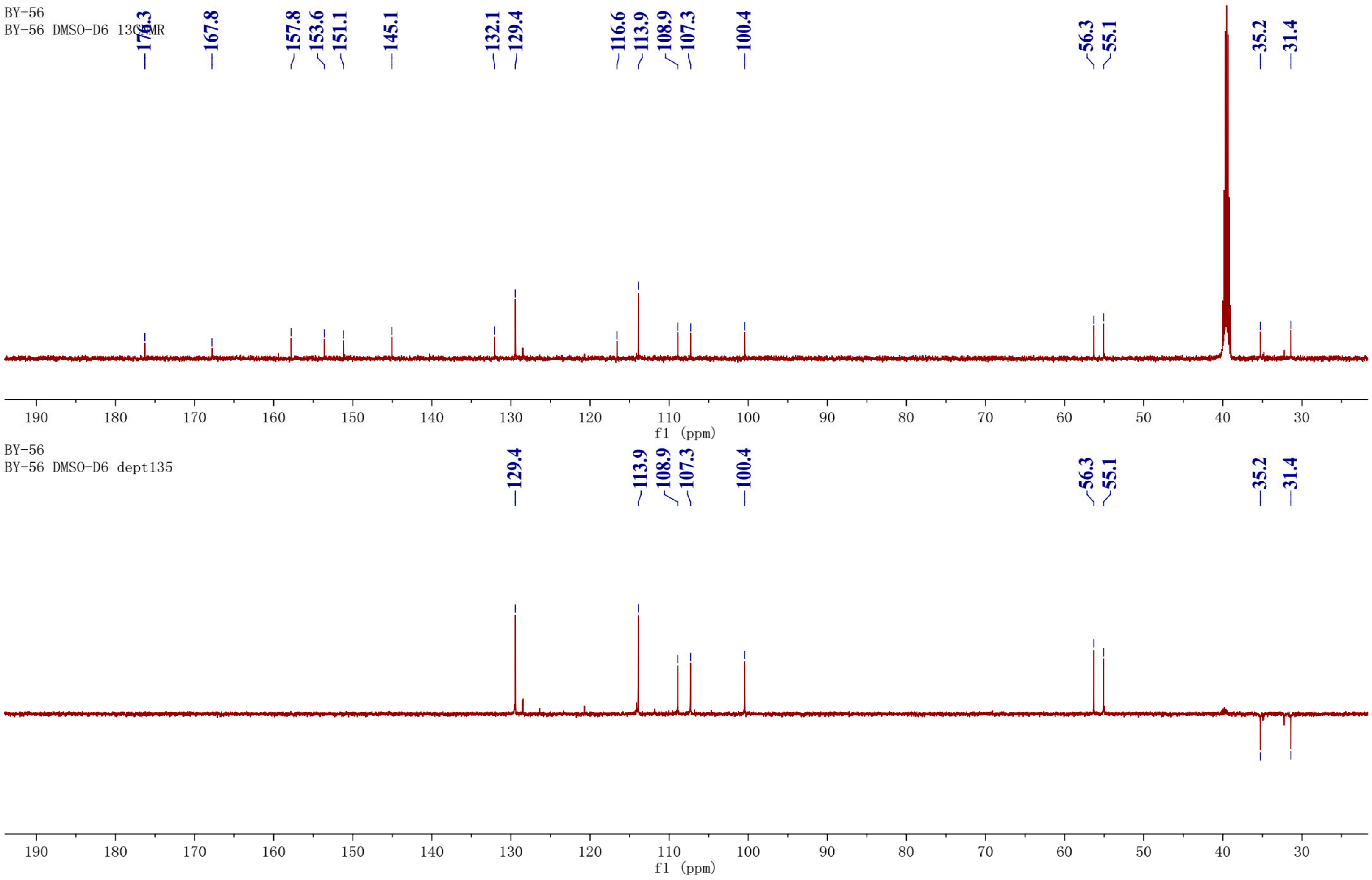


**Figure S10.** $^{13}C$-NMR spectrum (125 MHz) of compound **2** in DMSO.

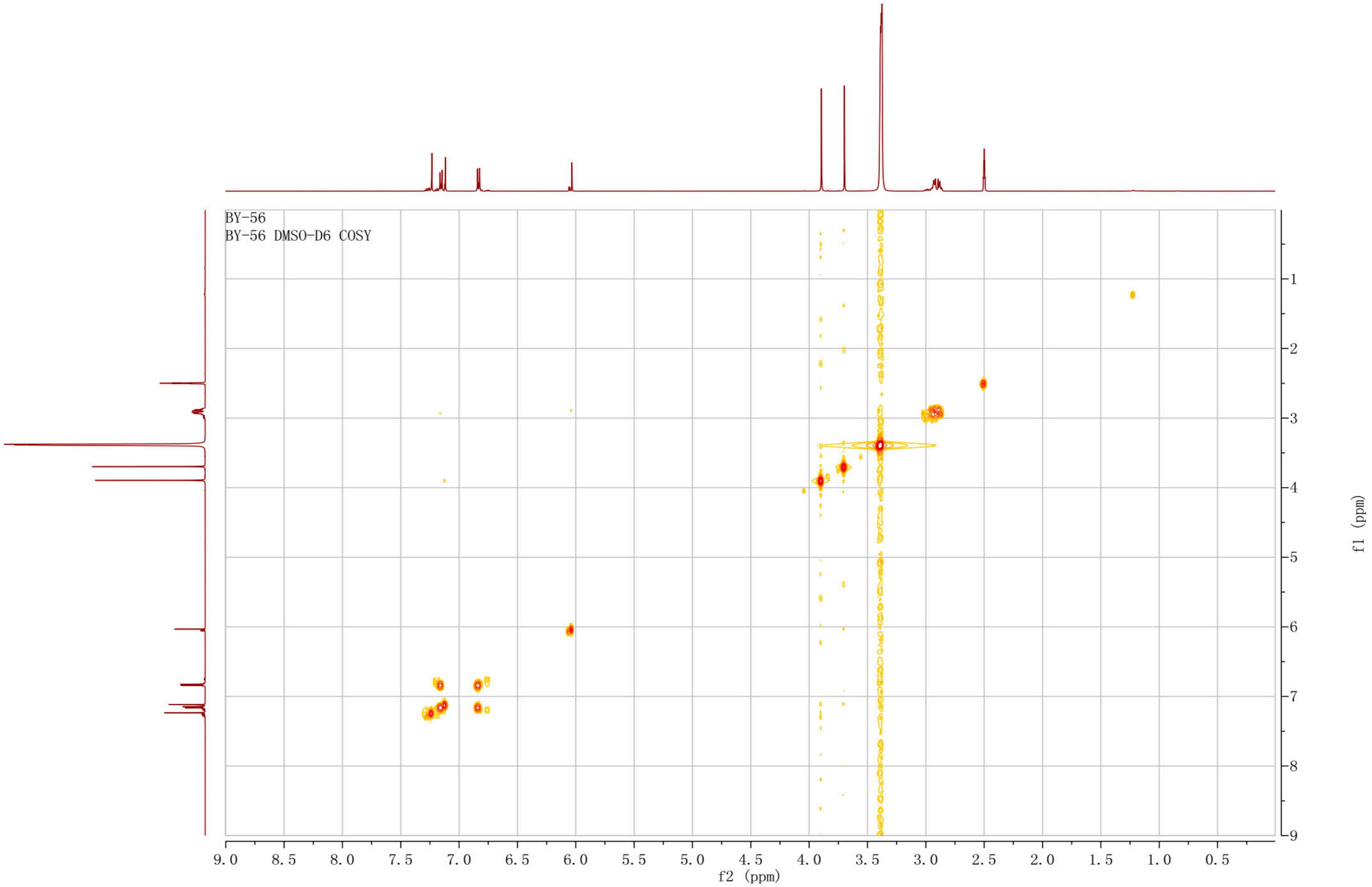


**Figure S11.** $^{1}H$-$^{1}H$ COSY spectrum (500 MHz) of compound **2** in DMSO.

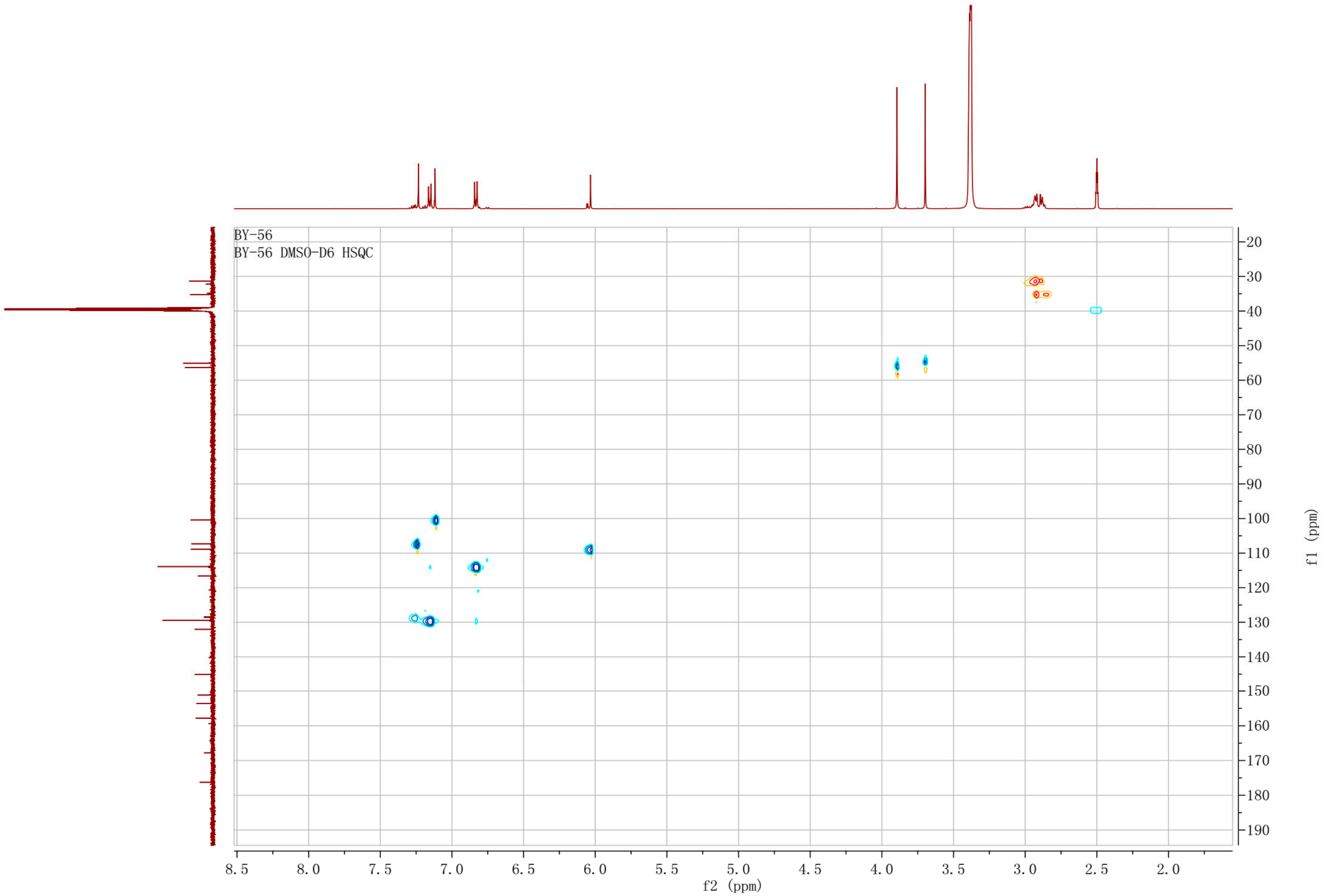


**Figure S12.** HSQC spectrum (500 MHz) of compound **2** in DMSO.

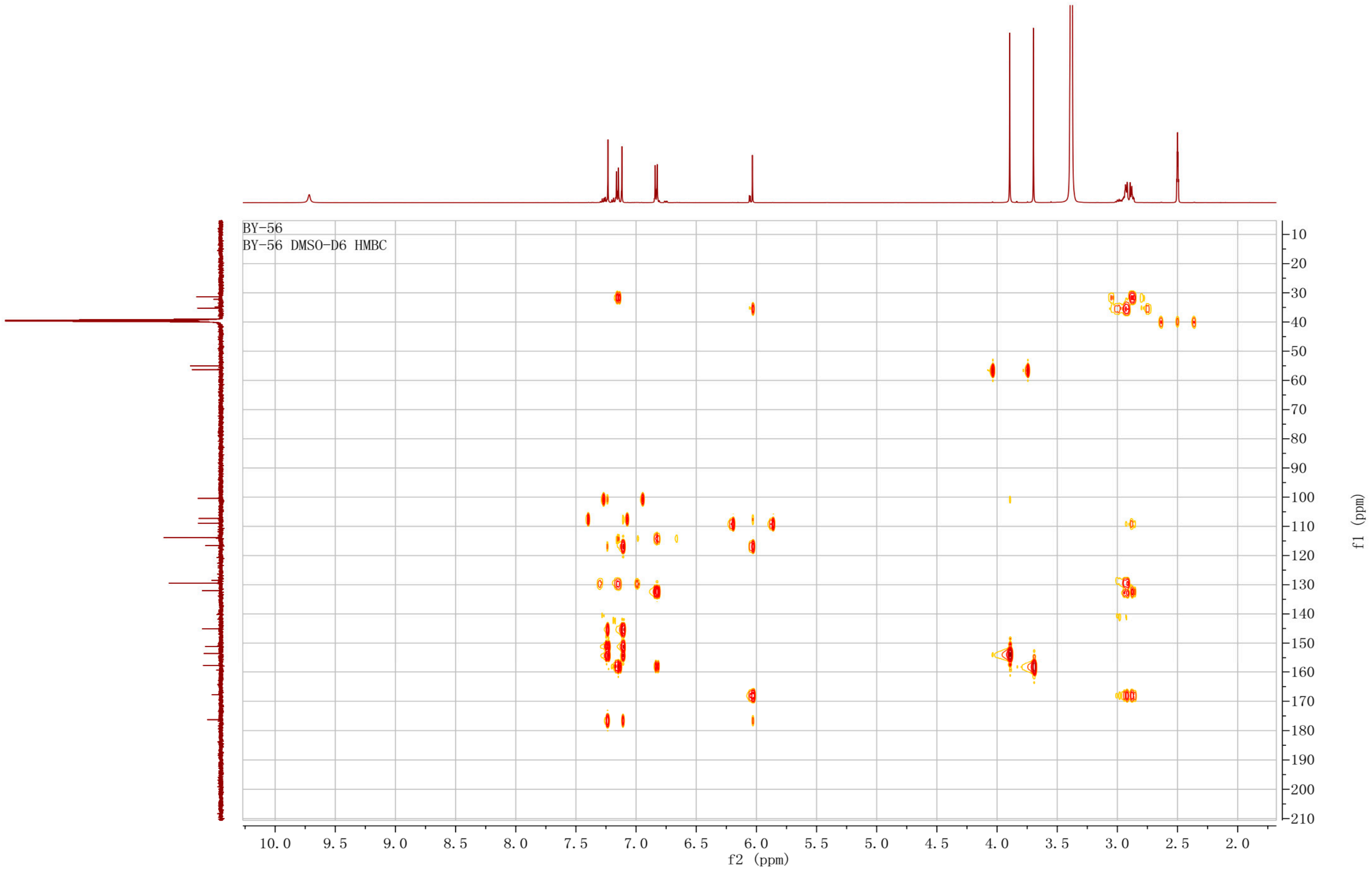


**Figure S13.** HMBC spectrum (500 MHz) of compound **2** in DMSO.

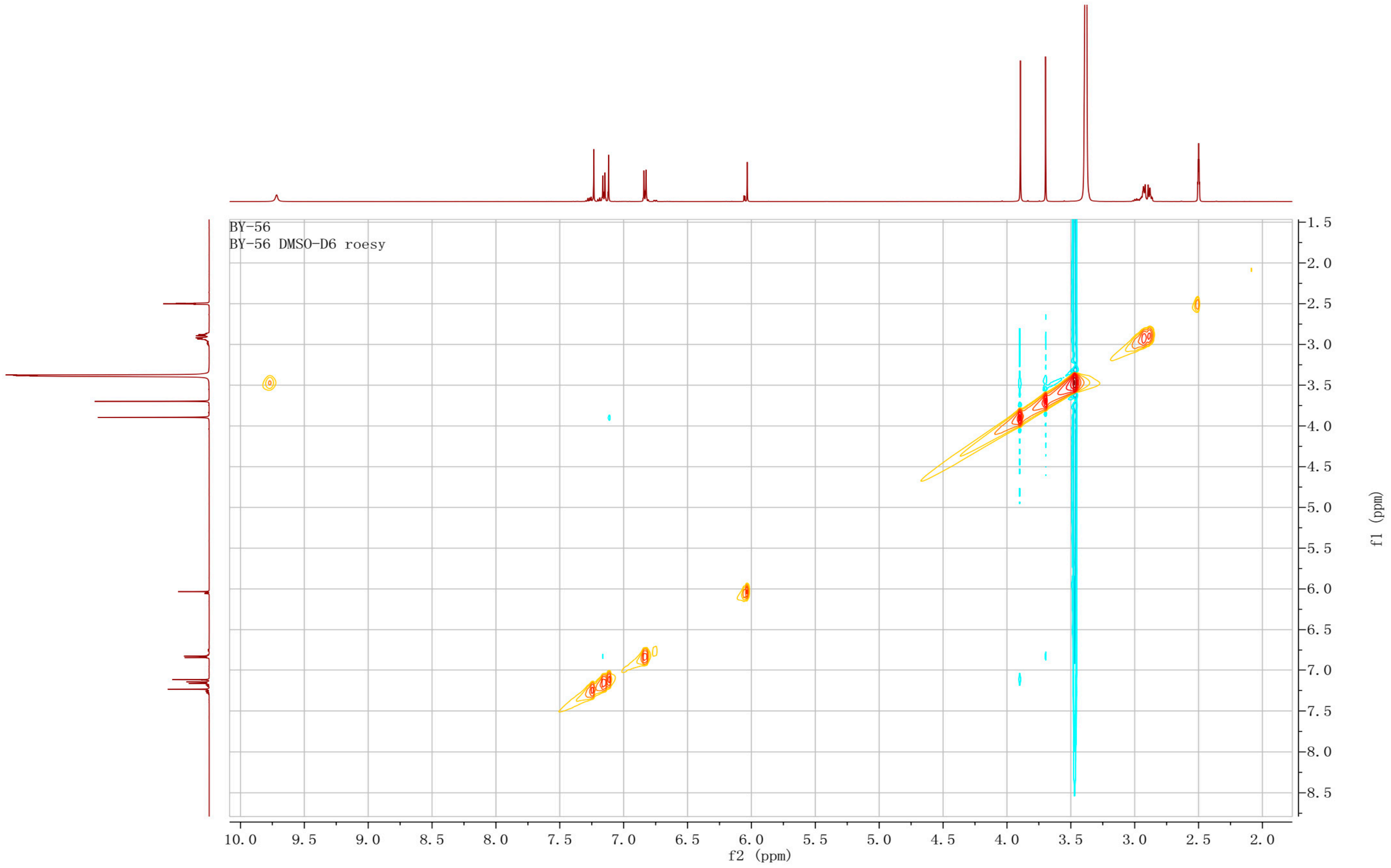


**Figure S14.** ROESY spectrum (400 MHz) of compound **2** in DMSO.

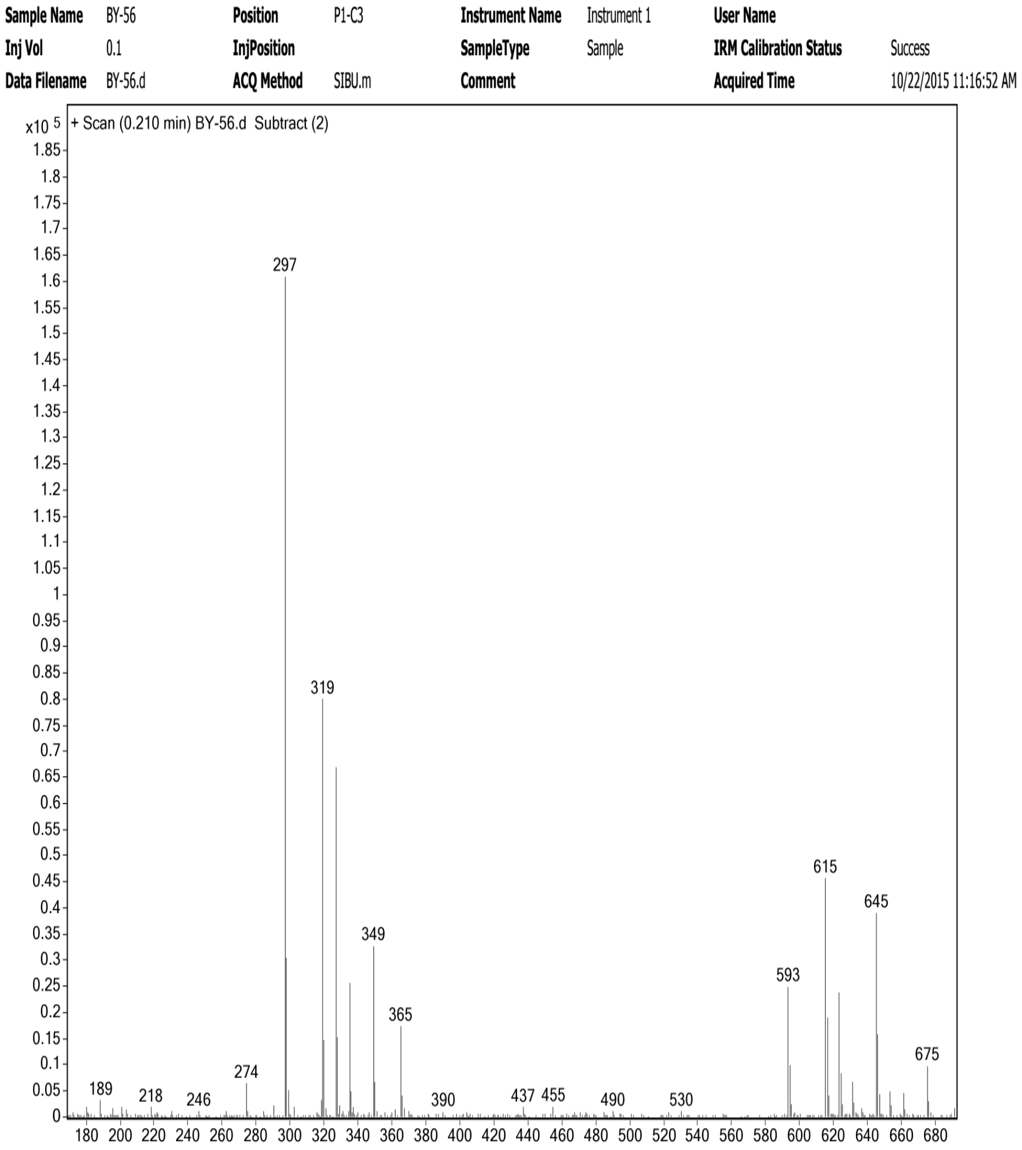


**Figure S15.** ESI(+)MS spectrum of compound **2**.

# Qualitative Analysis Report

| | | | |
|---|---|---|---|
| **Data Filename** | BY-56.d | **Sample Name** | BY-56 |
| **Sample Type** | Sample | **Position** | P1-C3 |
| **Instrument Name** | Instrument 1 | **User Name** | |
| **Acq Method** | SIBU.m | **Acquired Time** | 10/22/2015 11:16:52 AM |
| **IRM Calibration Status** | Success | **DA Method** | ESI+.m |
| **Comment** | | | |

**Sample Group** **Info.**
**Acquisition SW Version** 6200 series TOF/6500 series Q-TOF B.05.01 (B5125.2)

## User Spectra

| Fragmentor Voltage | Collision Energy | Ionization Mode |
|---|---|---|
| 135 | 0 | ESI |

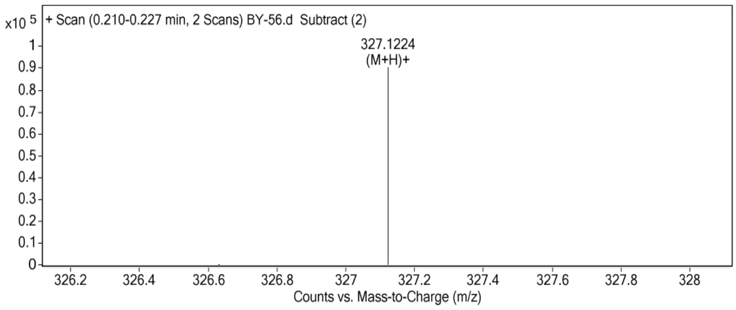


**Peak List**

| *m/z* | z | Abund | Formula | Ion |
|---|---|---|---|---|
| 297.1121 | 1 | 218337.25 | | |
| 319.094 | 1 | 102590.45 | | |
| 327.1224 | 1 | 90408.25 | C19 H18 O5 | (M+H)+ |
| 349.1047 | 1 | 43308.2 | | |
| 593.2171 | 1 | 49472.85 | | |
| 615.1992 | 1 | 108167.98 | | |
| 623.2275 | 1 | 45127.51 | | |
| 645.2098 | 1 | 93037.04 | | |

**Formula Calculator Element Limits**

| Element | Min | Max |
|---|---|---|
| C | 3 | 60 |
| H | 0 | 120 |
| O | 0 | 30 |
| N | 0 | 30 |

**Formula Calculator Results**

| Formula | CalculatedMass | CalculatedMz | Mz | Diff. (mDa) | Diff. (ppm) | DBE |
|---|---|---|---|---|---|---|
| C19 H18 O5 | 326.1154 | 327.1227 | 327.1224 | 0.3 | 1.0 | 11.0000 |

--- End Of Report ---

Agilent Technologies Page 1 of 1 Printed at: 2:32 PM on: 10/22/2015

**Figure S16.** HRESI(+)MS spectrum of compound **2**.

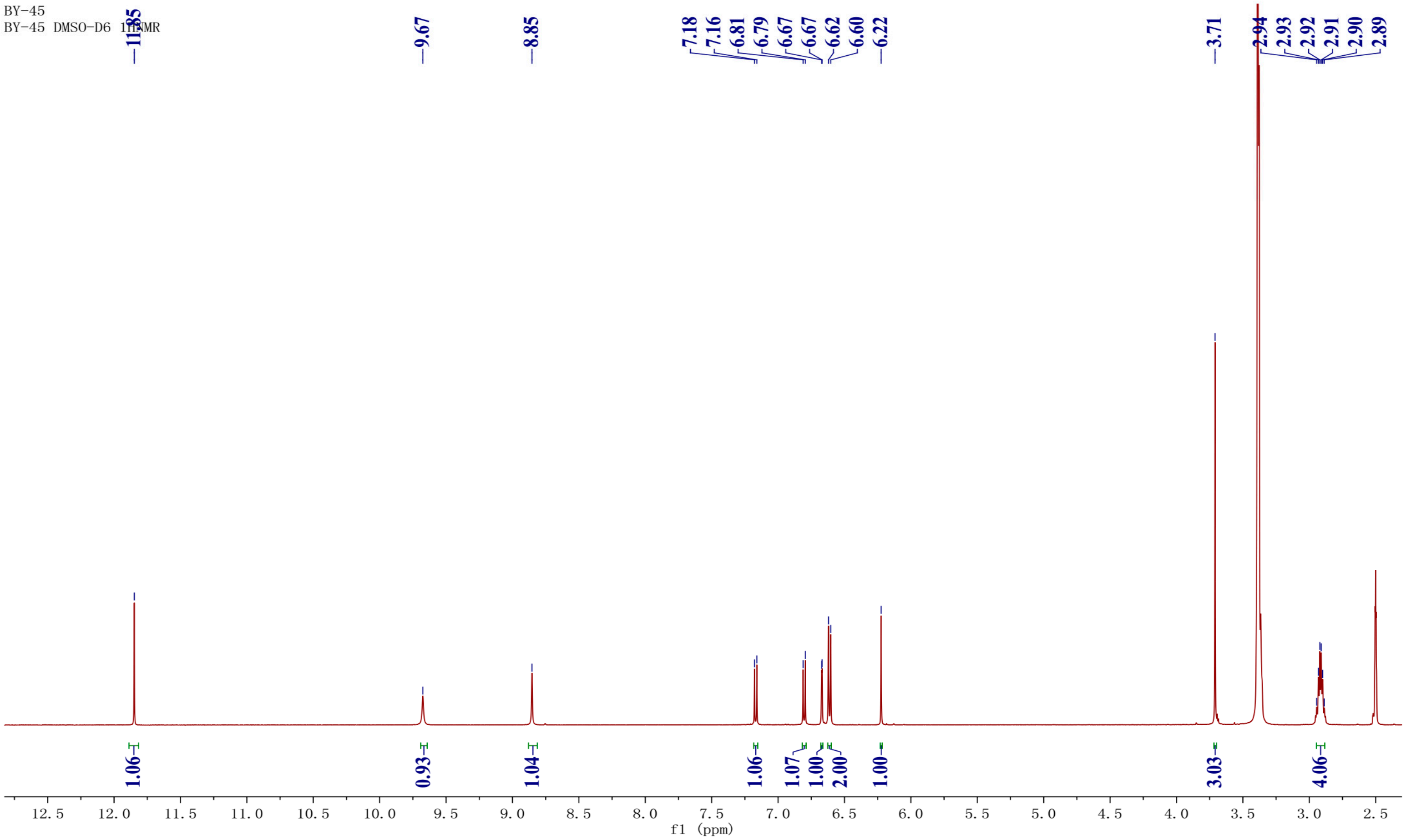


**Figure S17.** $^{1}$H-NMR spectrum (500 MHz) of compound **3** in DMSO.

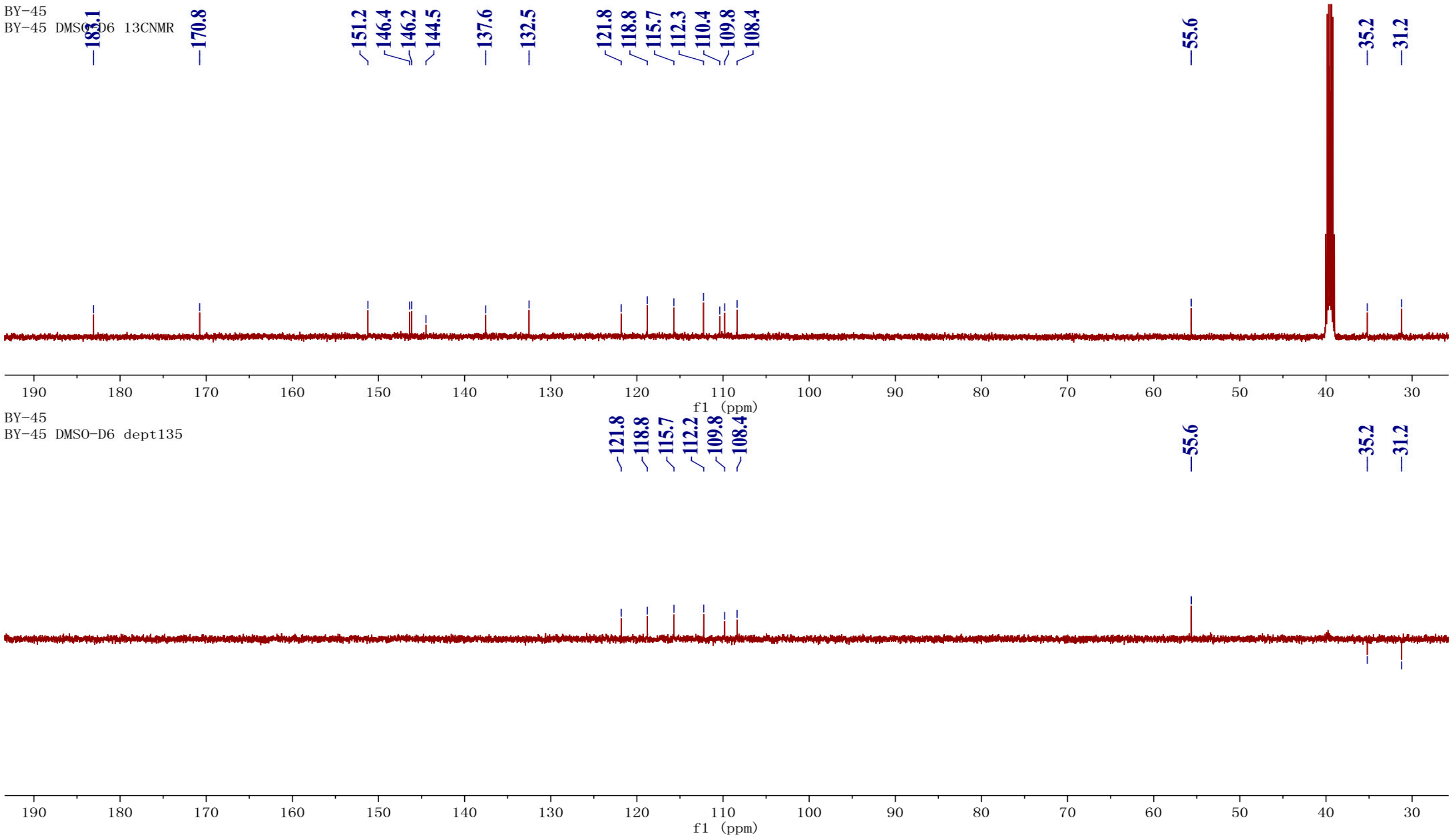


**Figure S18.** $^{13}$C-NMR spectrum (125 MHz) of compound **3** in DMSO.

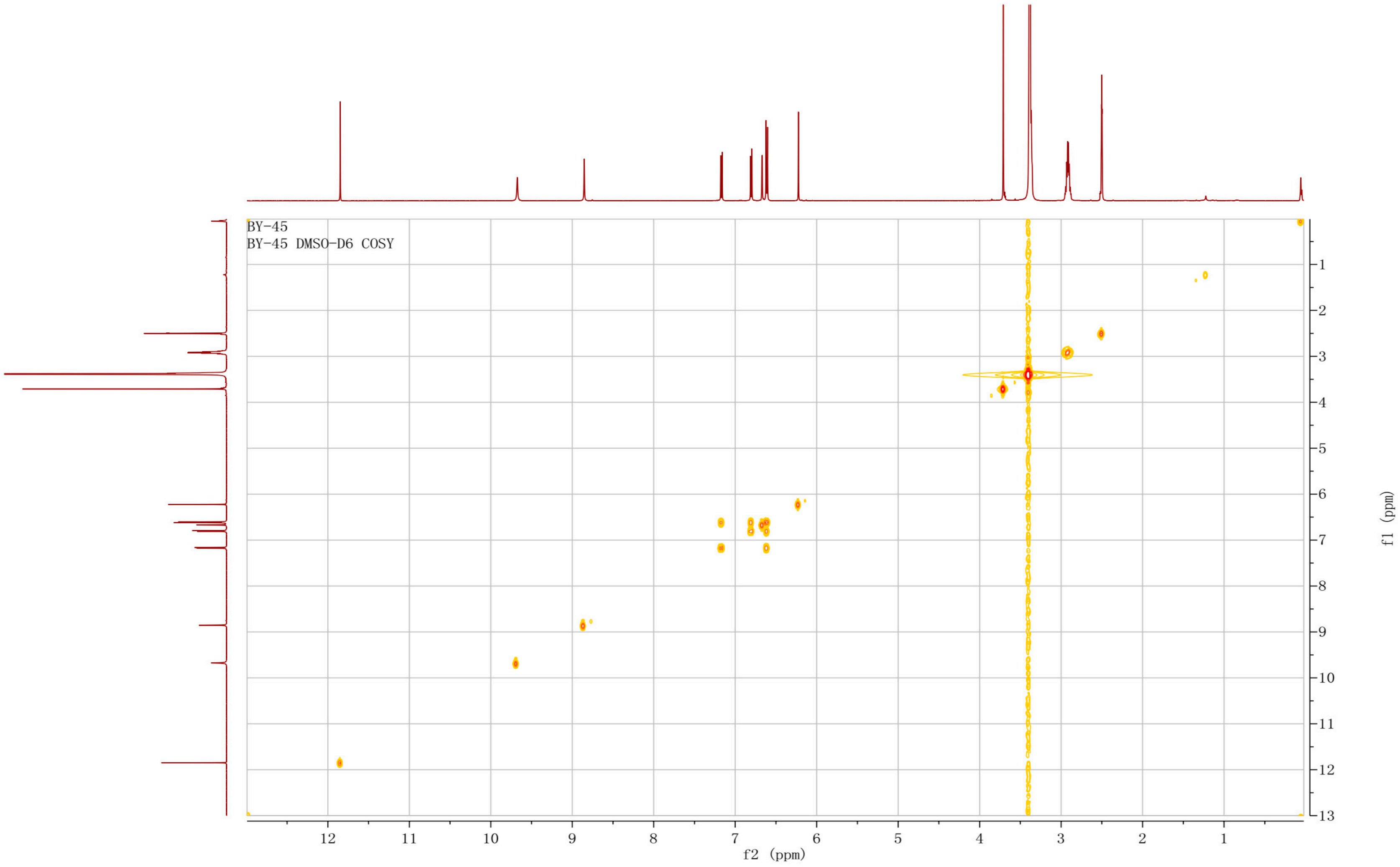


**Figure S19.** $^1$H-$^1$H COSY spectrum (500 MHz) of compound **3** in DMSO.

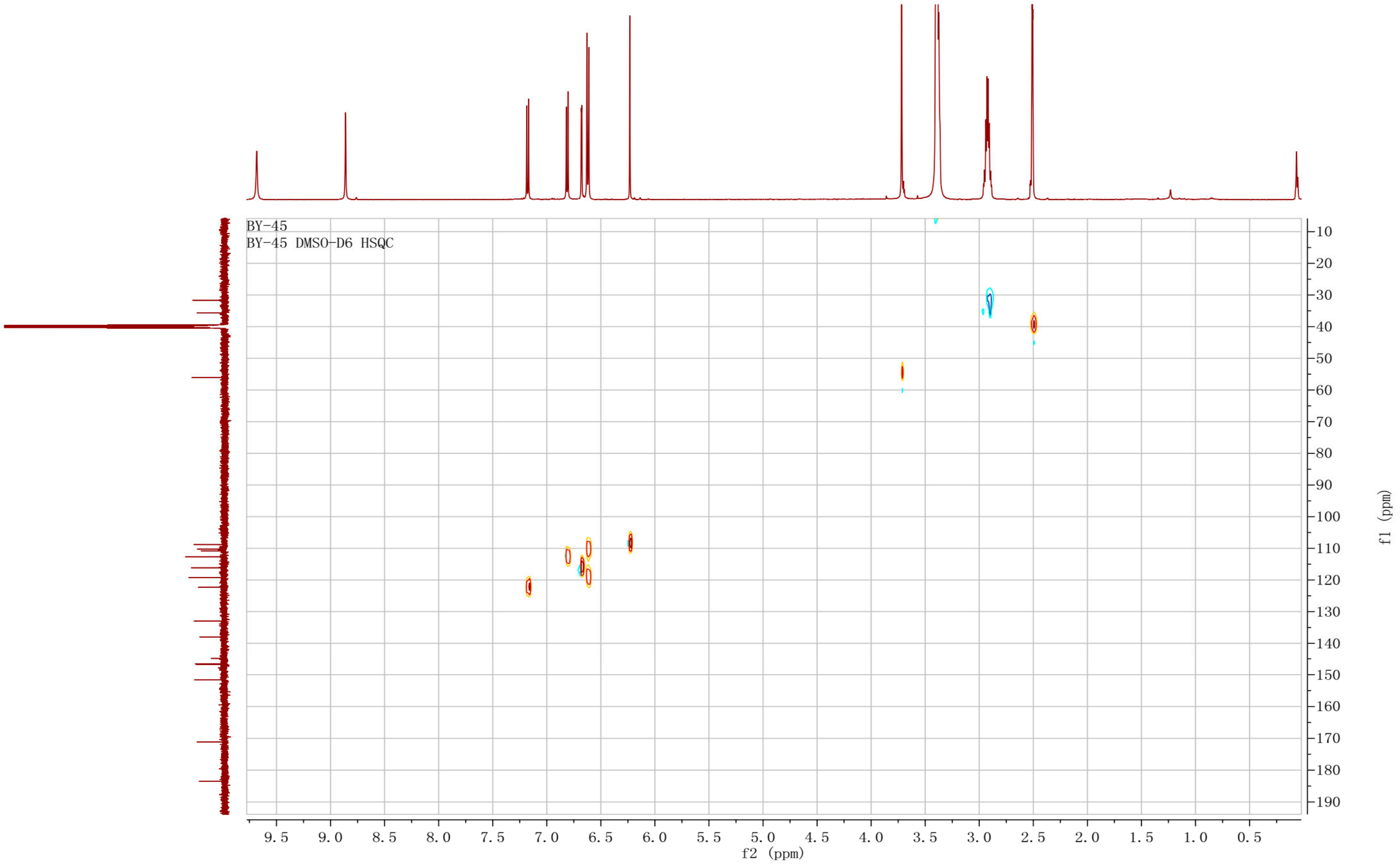


**Figure S20.** HSQC spectrum (500 MHz) of compound **3** in DMSO.

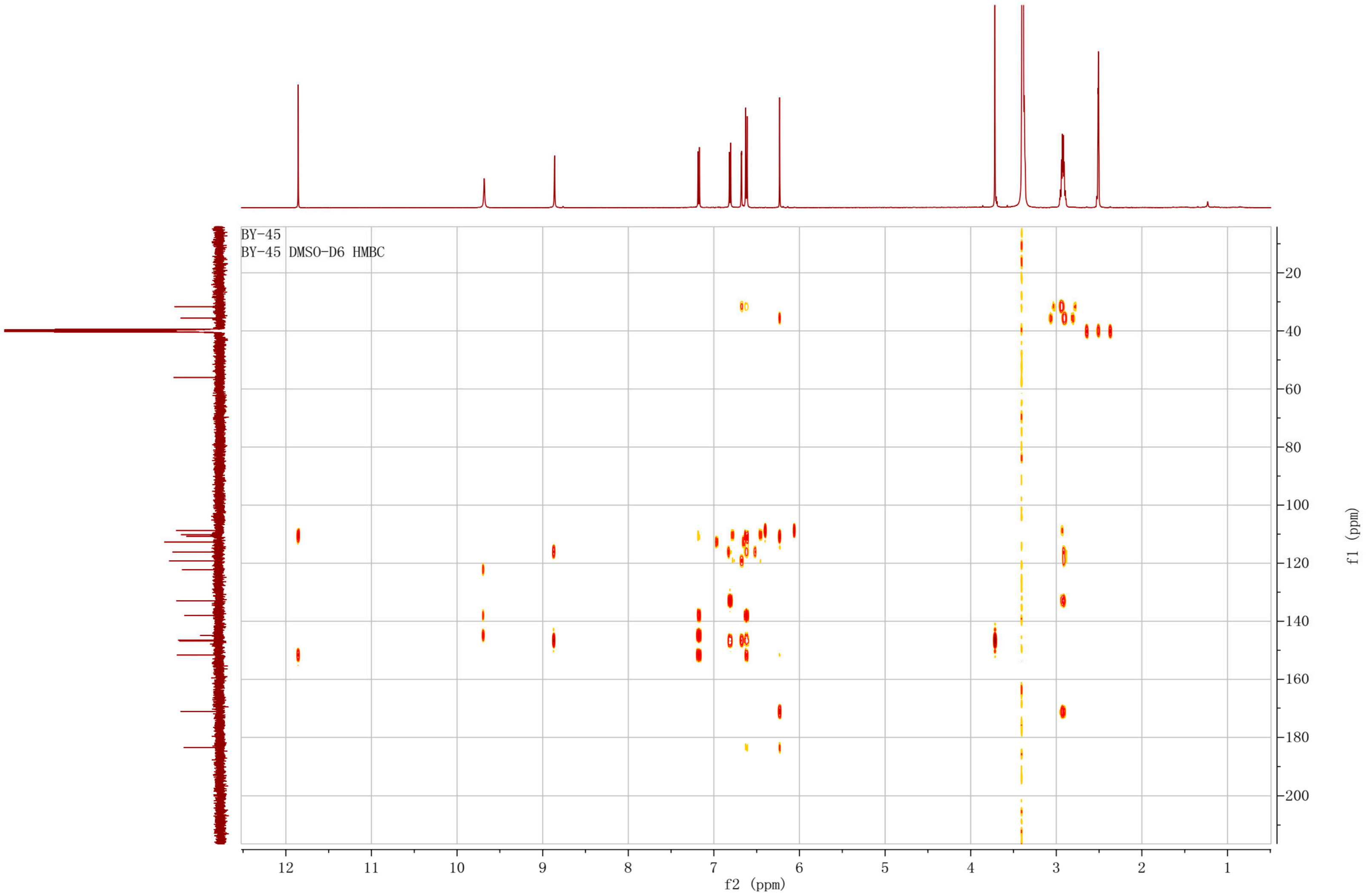


**Figure S21.** HMBC spectrum (500 MHz) of compound **3** in DMSO.

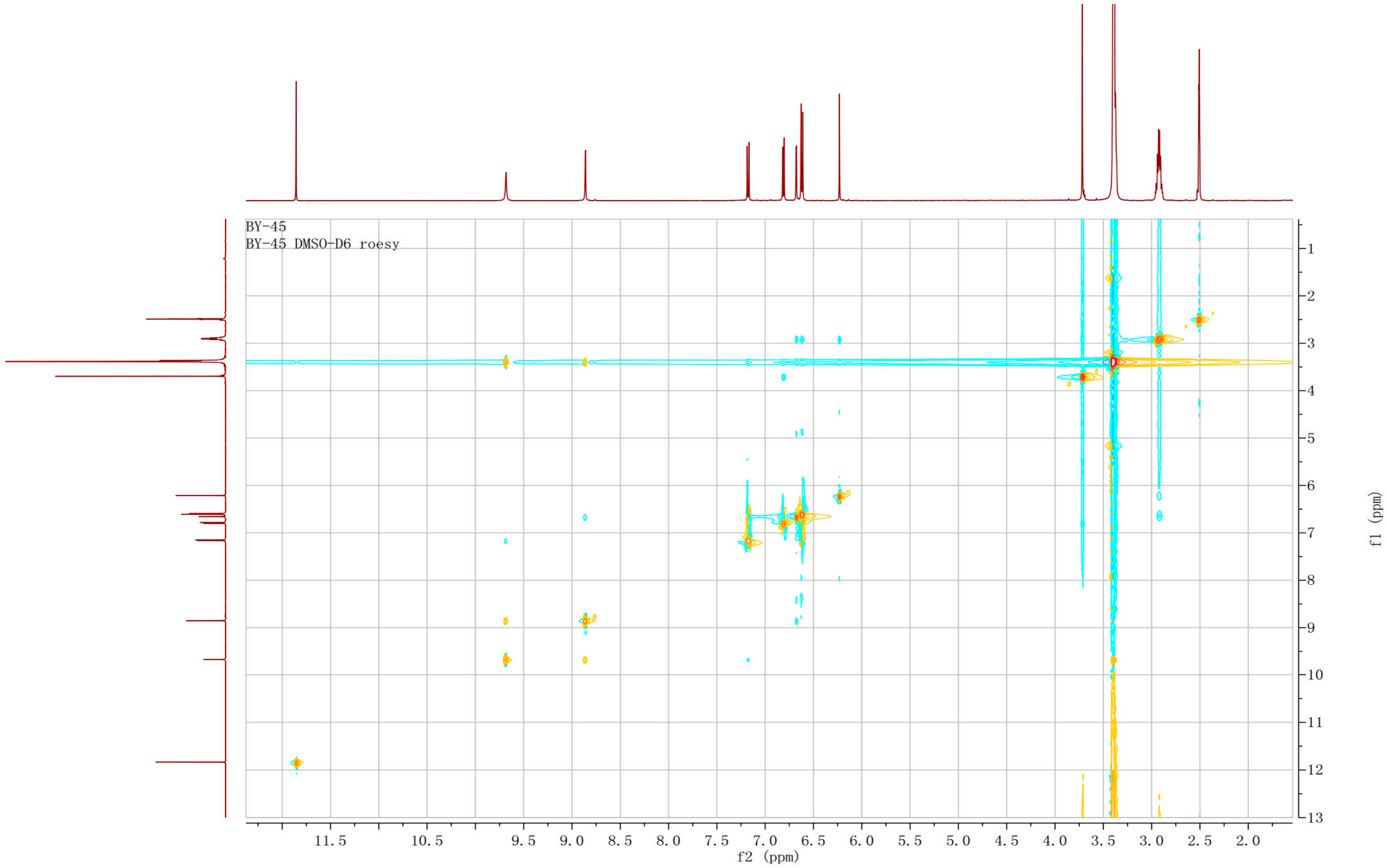


**Figure S22.** ROESY spectrum (400 MHz) of compound **3** in DMSO.

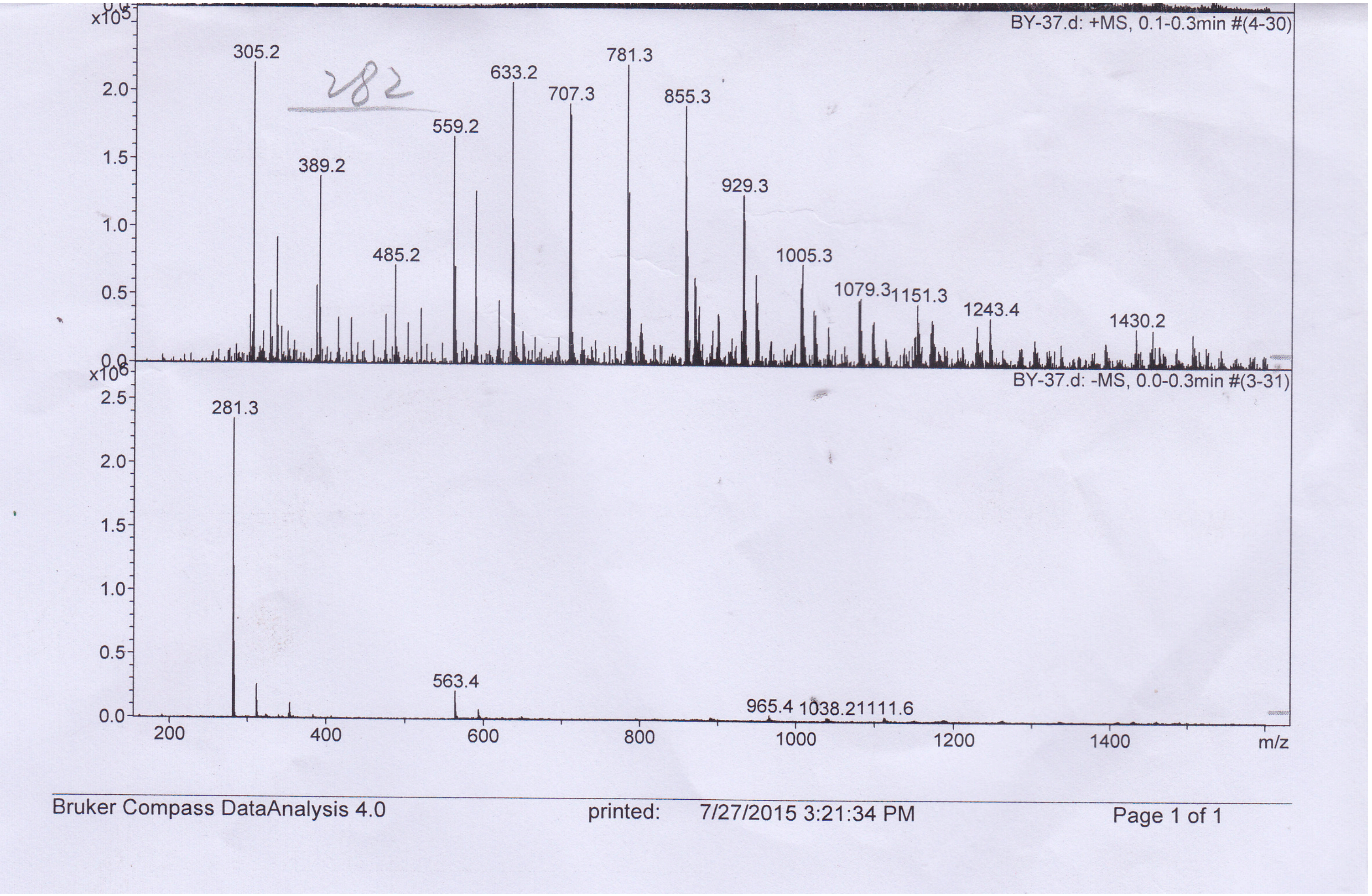


**Figure S23.** ESI(+)MS spectrum of compound **3**.

# Qualitative Analysis Report

| | | | |
|---|---|---|---|
| **Data Filename** | BY-45-.d | **Sample Name** | BY-45 |
| **Sample Type** | Sample | **Position** | P1-C2 |
| **Instrument Name** | Instrument 1 | **User Name** | |
| **Acq Method** | SIBU-ESI-i.m | **Acquired Time** | 10/22/2015 3:20:12 PM |
| **IRM Calibration Status** | Success | **DA Method** | ESI+.m |
| **Comment** | | | |

**Sample Group** **Info.**
**Acquisition SW Version** 6200 series TOF/6500 series Q-TOF B.05.01 (B5125.2)

## User Spectra

| Fragmentor Voltage | Collision Energy | Ionization Mode |
|---|---|---|
| 135 | 0 | ESI |

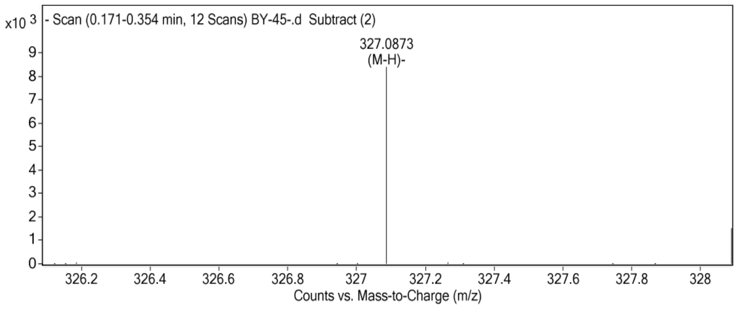


**Peak List**

| *m/z* | z | Abund | Formula | Ion |
|---|---|---|---|---|
| 89.0246 | | 1365.38 | | |
| 327.0873 | 1 | 8401.06 | C18 H16 O6 | (M-H)- |
| 328.0908 | 1 | 1529.88 | C18 H16 O6 | (M-H)- |
| 390.0831 | 1 | 1013.56 | | |
| 411.0692 | 1 | 706.99 | | |
| 441.08 | 1 | 19259.49 | | |
| 442.0848 | 1 | 3664.01 | | |
| 443.0859 | 1 | 724.34 | | |

**Formula Calculator Element Limits**

| Element | Min | Max |
|---|---|---|
| C | 3 | 60 |
| H | 0 | 120 |
| O | 0 | 30 |
| N | 0 | 5 |

**Formula Calculator Results**

| Formula | CalculatedMass | CalculatedMz | Mz | Diff. (mDa) | Diff. (ppm) | DBE |
|---|---|---|---|---|---|---|
| C18 H16 O6 | 328.0947 | 327.0874 | 327.0873 | -0.1 | -0.3 | 11.0000 |

--- End Of Report ---



**Figure S24.** HRESI(+)MS spectrum of compound **3**.

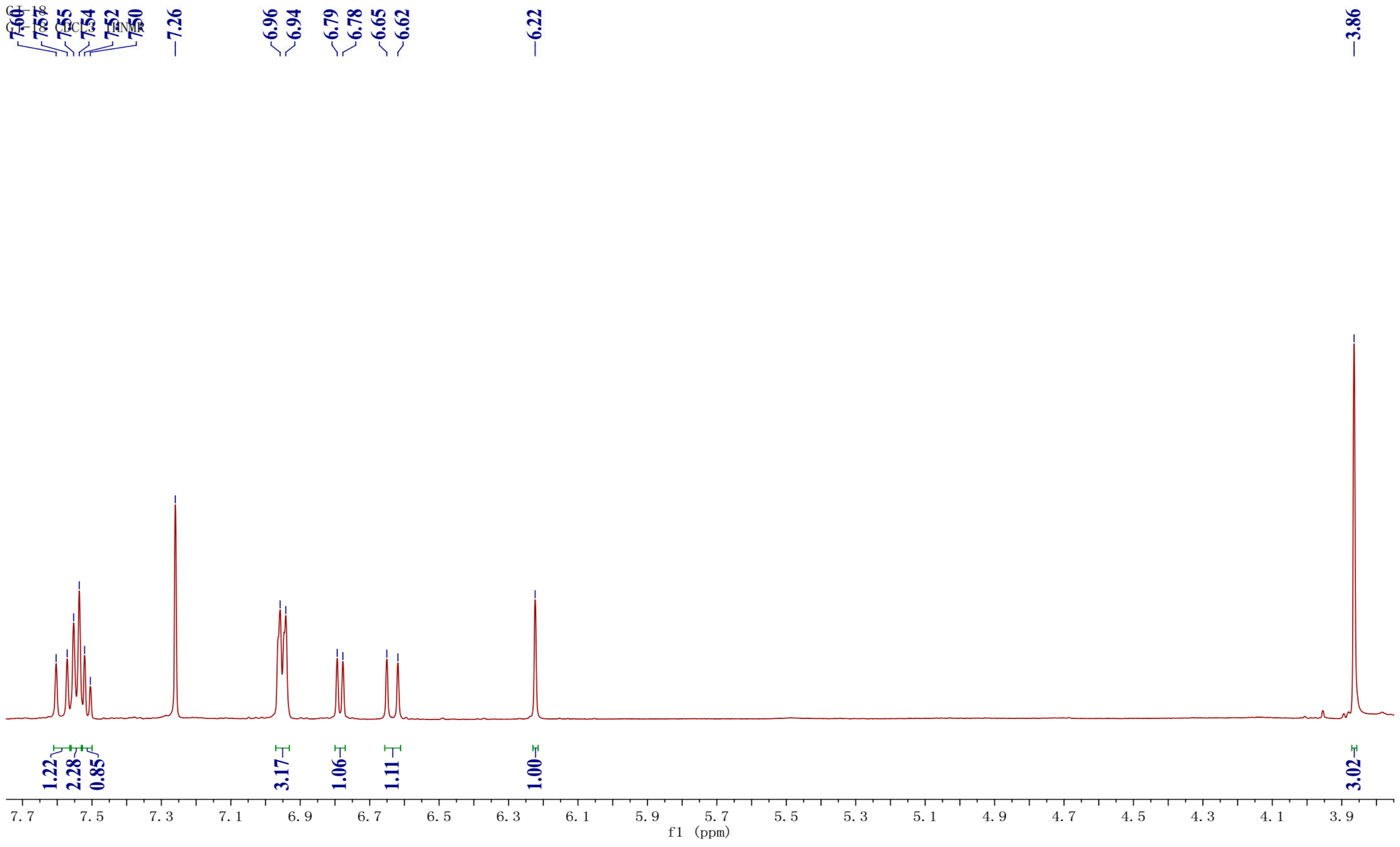

**Figure S25.** $^{1}$H-NMR spectrum (500 MHz) of compound **4** in $CDCl_3$.

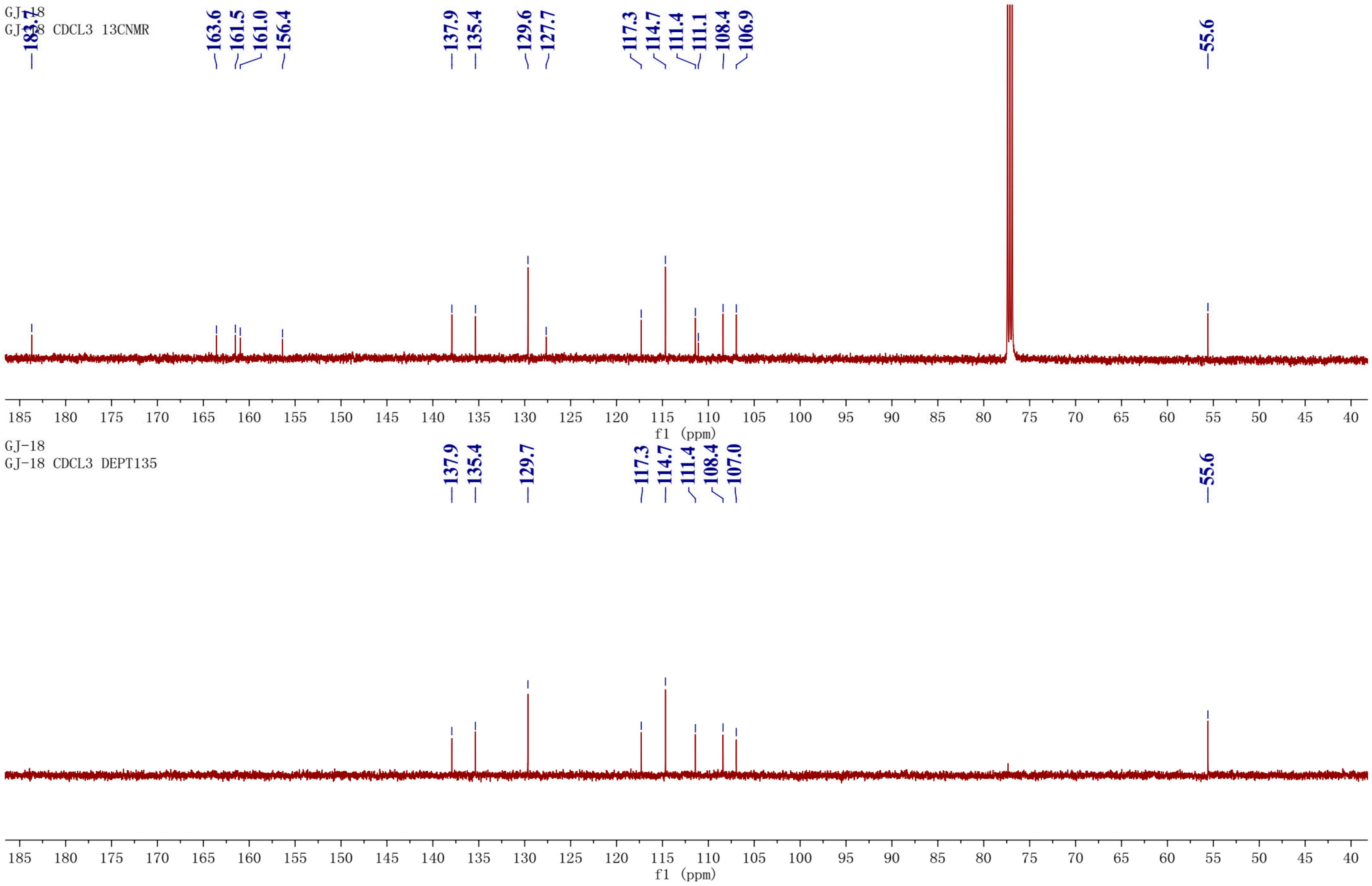


**Figure S26.** $^{13}C$-NMR spectrum (125 MHz) of compound **4** in $CDCl_3$.

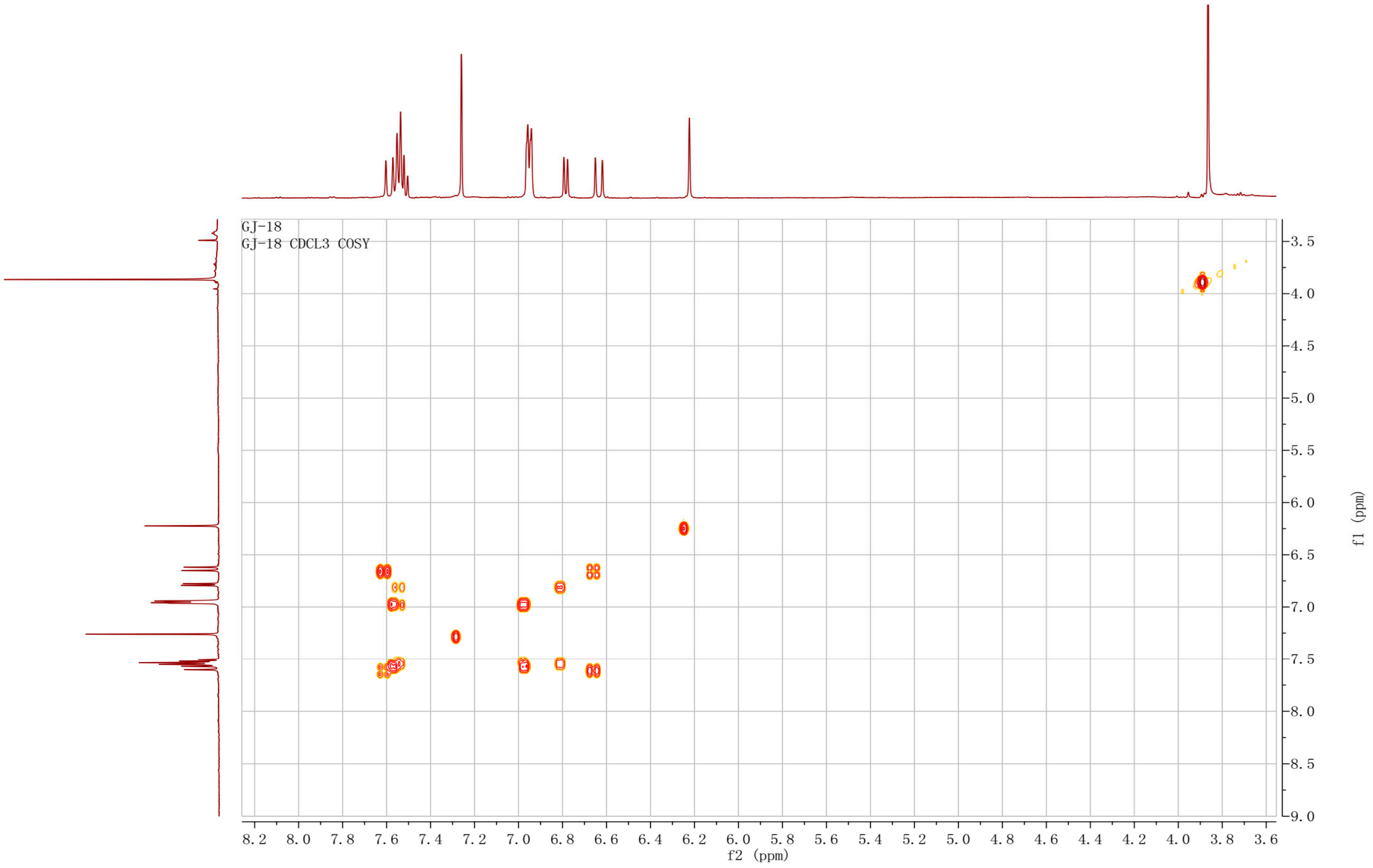


**Figure S27.** $^{1}H$-$^{1}H$ COSY spectrum (500 MHz) of compound **4** in $CDCl_3$.

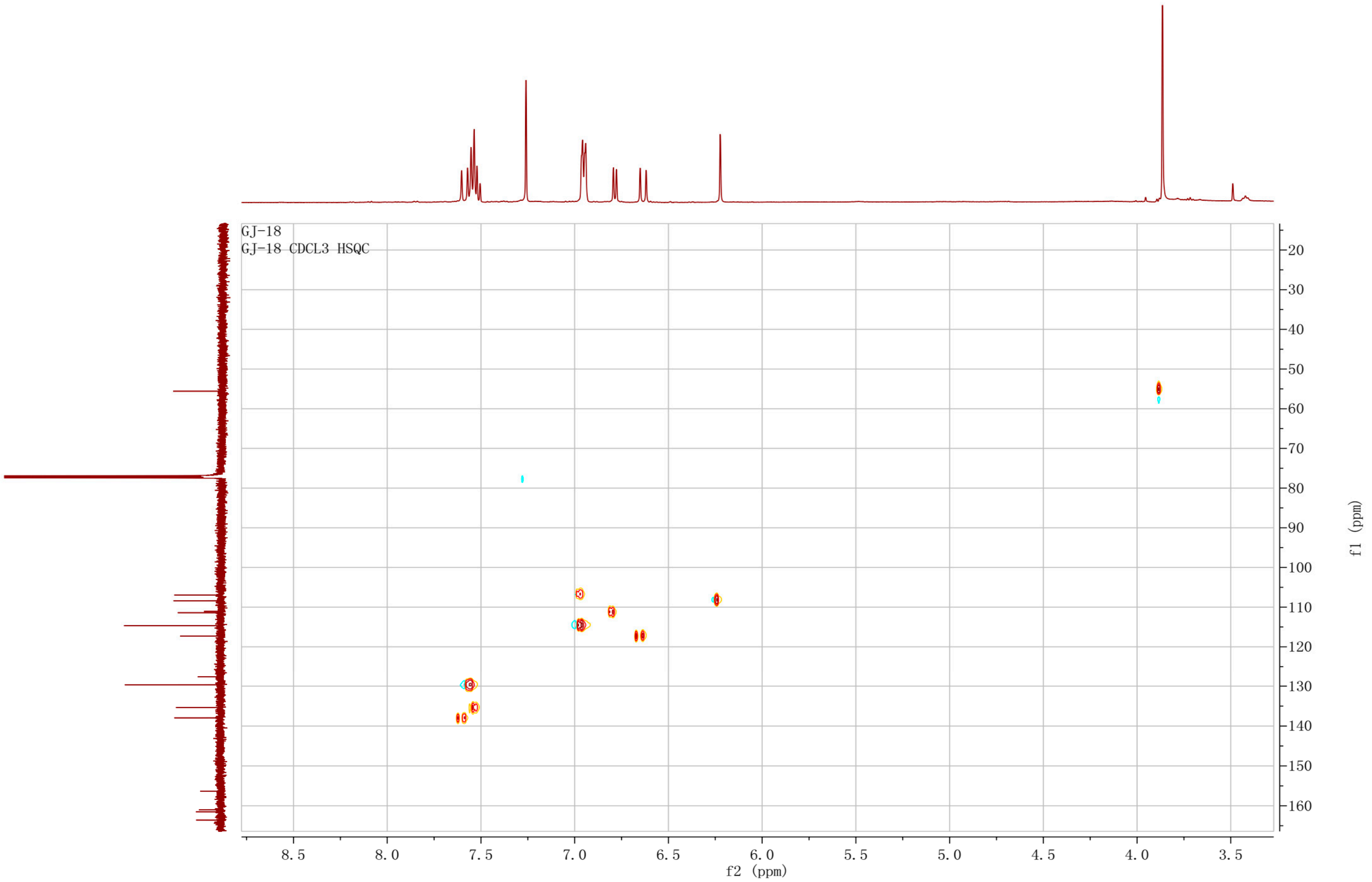


**Figure S28.** HSQC spectrum (500 MHz) of compound **4** in $CDCl_3$.

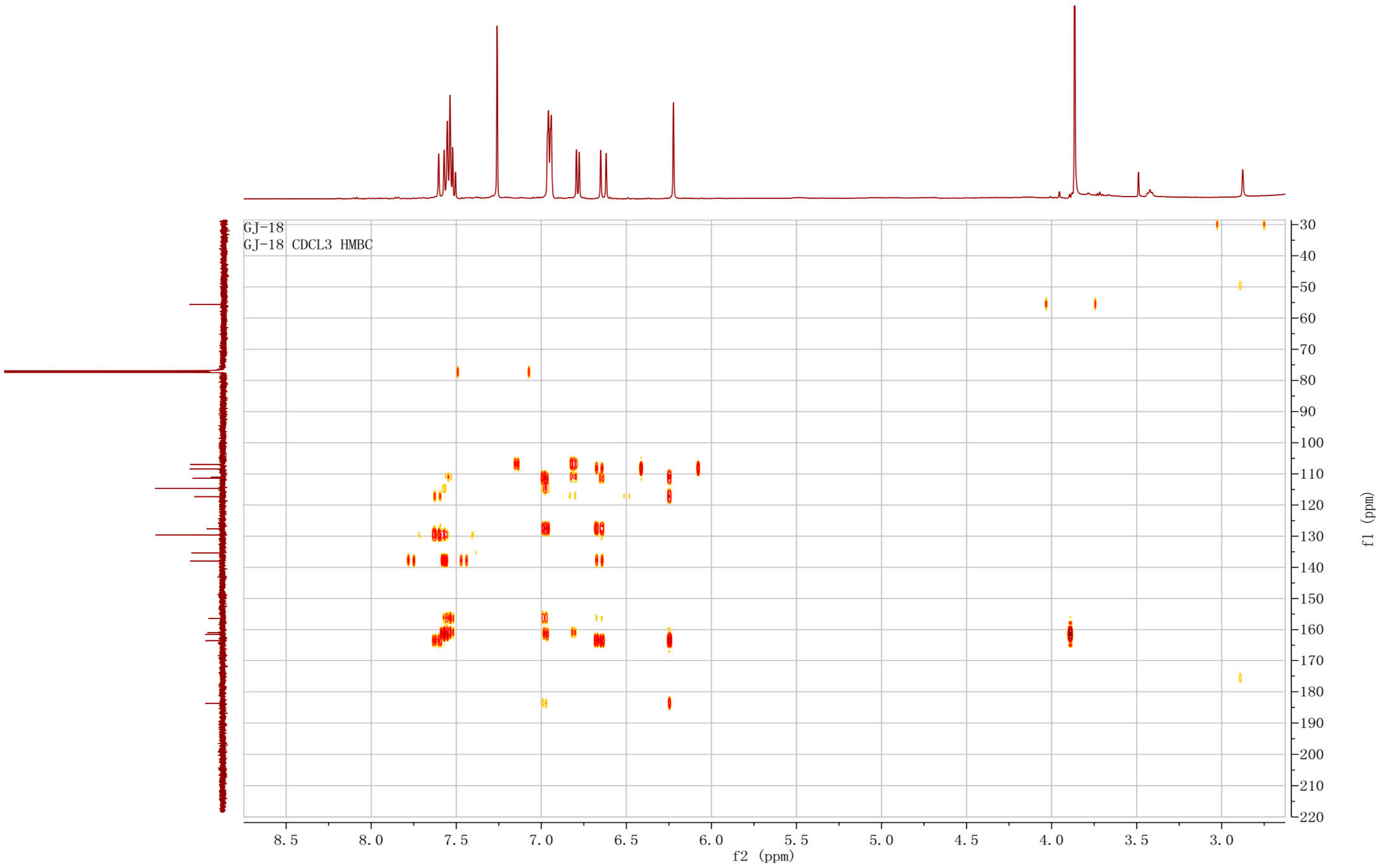


**Figure S29.** HMBC spectrum (500 MHz) of compound **4** in $CDCl_3$.

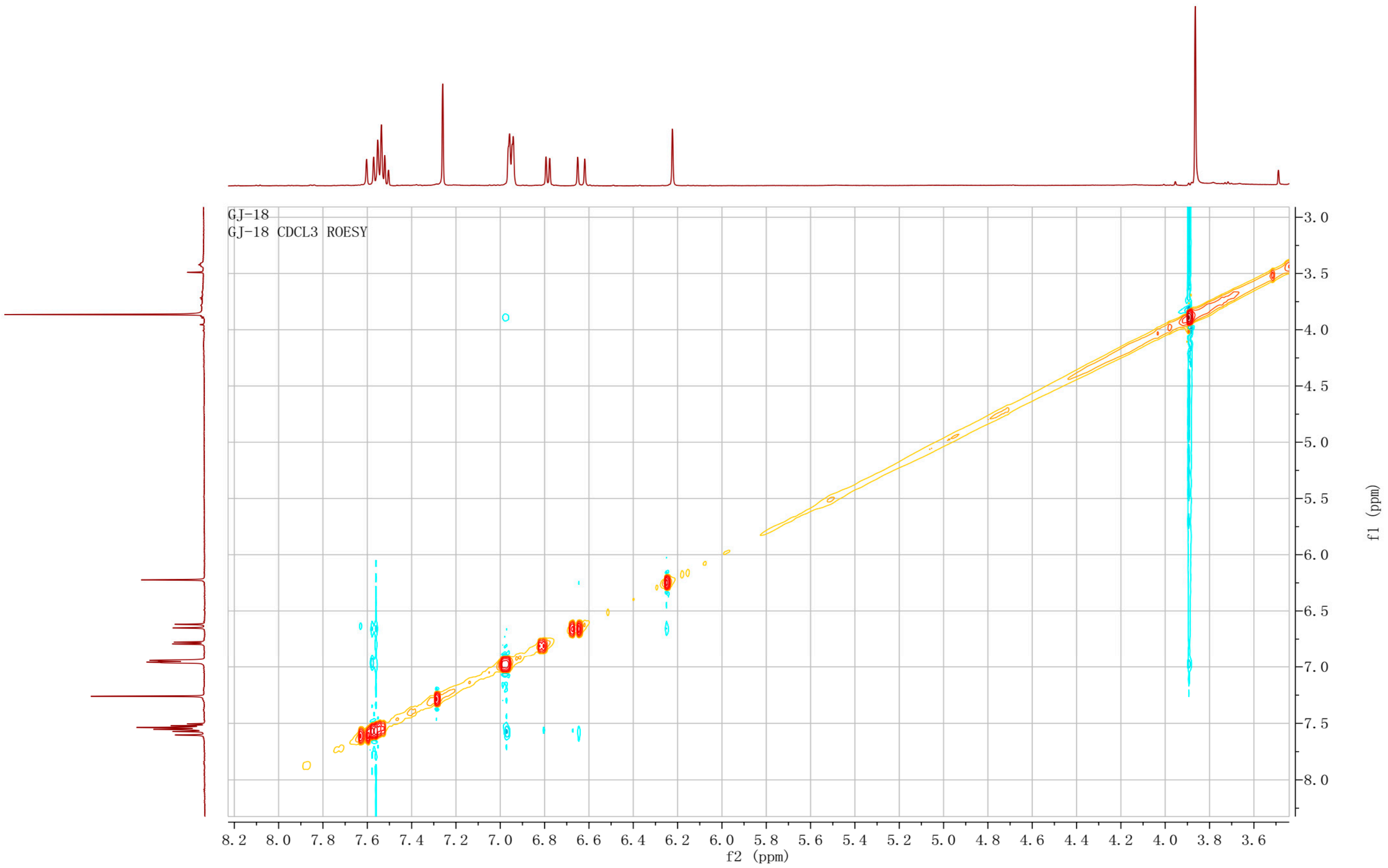


**Figure S30.** ROESY spectrum (400 MHz) of compound **4** in $CDCl_3$.

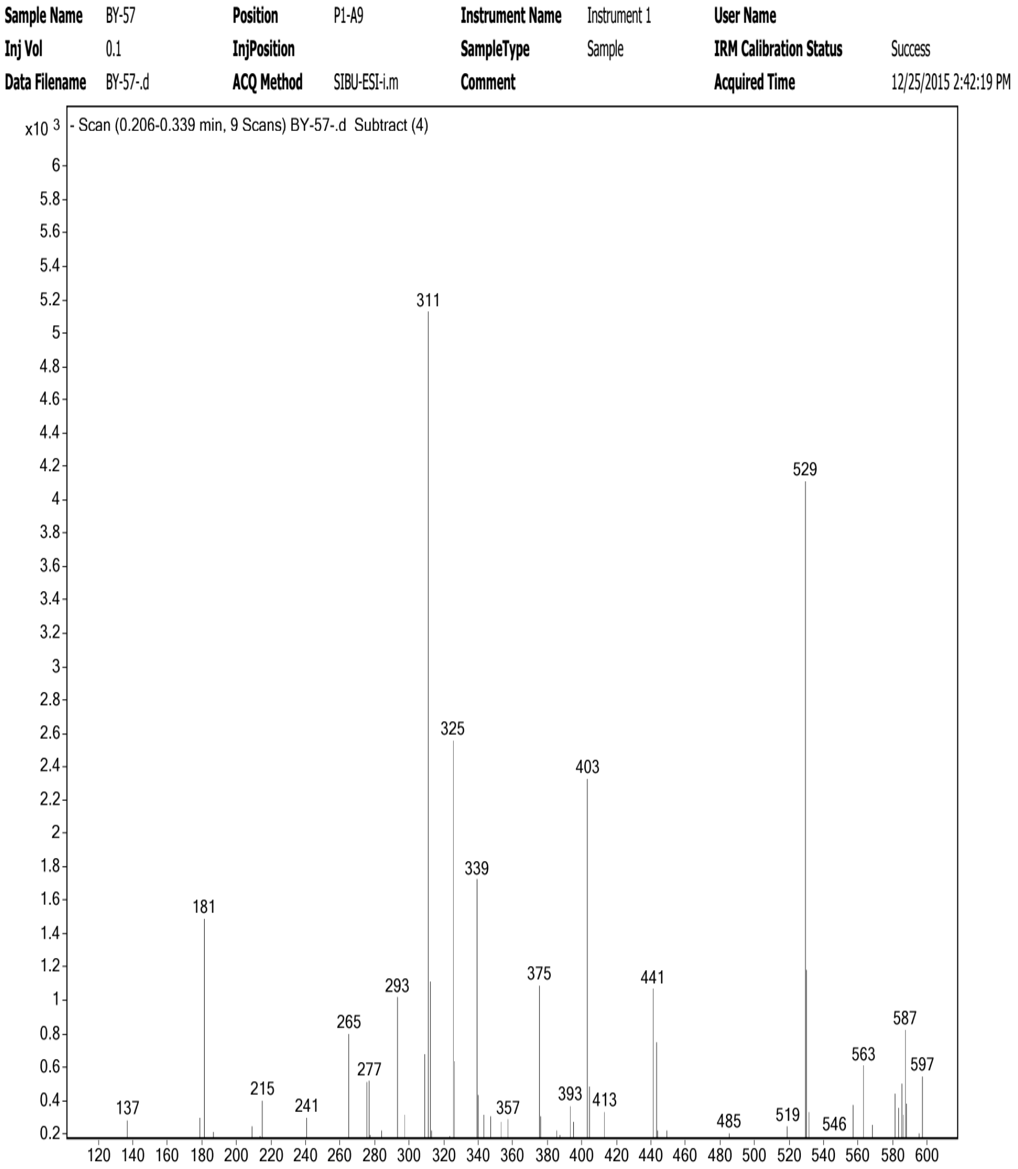


**Figure S31.** ESI(+)MS spectrum of compound **4**.

# Qualitative Analysis Report

| | | | |
|---|---|---|---|
| **Data Filename** | BY-57-.d | **Sample Name** | BY-57 |
| **Sample Type** | Sample | **Position** | P1-A9 |
| **Instrument Name** | Instrument 1 | **User Name** | |
| **Acq Method** | SIBU-ESI-i.m | **Acquired Time** | 12/25/2015 3:05:43 PM |
| **IRM Calibration Status** | Success | **DA Method** | ESI+.m |
| **Comment** | | | |

**Sample Group** **Info.**
**Acquisition SW** 6200 series TOF/6500 series
**Version** Q-TOF B.05.01 (B5125.2)

## User Spectra

| Fragmentor Voltage | Collision Energy | Ionization Mode |
|---|---|---|
| 135 | 0 | ESI |

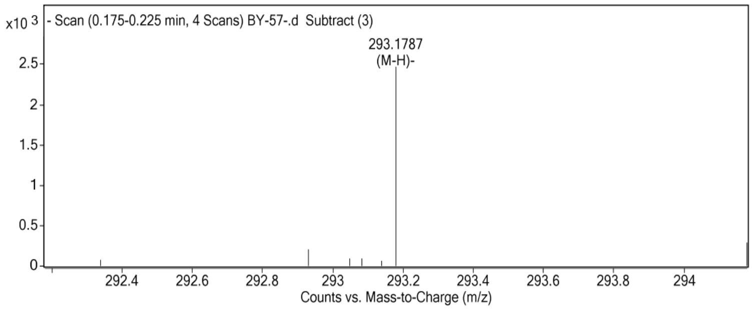


**Peak List**

| *m/z* | z | Abund |
|---|---|---|
| 311.1687 | 1 | 5084.09 |
| 325.1844 | 1 | 6385.03 |
| 339.2001 | 1 | 4304.08 |
| 375.2752 | 1 | 4148.44 |
| 403.3066 | 1 | 6881.47 |
| 441.2495 | 1 | 3392.89 |
| 529.2812 | 1 | 12609.48 |
| 530.2839 | 1 | 4065.03 |

**Formula Calculator Element Limits**

| Element | Min | Max |
|---|---|---|
| C | 3 | 120 |
| H | 0 | 240 |
| O | 0 | 60 |
| N | 0 | 10 |

**Formula Calculator Results**

| Formula | CalculatedMass | CalculatedMz | Mz | Diff. (mDa) | Diff. (ppm) | DBE |
|---|---|---|---|---|---|---|
| C20 H24 N O | 294.1858 | 293.1785 | 293.1787 | 0.0 | -0.1 | 9.5000 |

--- End Of Report ---

Agilent Technologies Page 1 of 1 Printed at: 3:09 PM on: 12/25/2015

**Figure S32.** HRESI(+)MS spectrum of compound **4**.
